# Coordination of Rapid Sphingolipid Responses to Heat Stress in Yeast

**DOI:** 10.1371/journal.pcbi.1003078

**Published:** 2013-05-30

**Authors:** Po-Wei Chen, Luis L. Fonseca, Yusuf A. Hannun, Eberhard O. Voit

**Affiliations:** 1 Integrative BioSystems Institute and Wallace H. Coulter Department of Biomedical Engineering, Georgia Institute of Technology, Atlanta, Georgia, United States of America; 2 Instituto de Tecnologia Quıímica e Biológica, Universidade Nova de Lisboa, Estação Agronómica Nacional, Oeiras, Portugal; 3 The Cancer Center at Stony Brook Medicine, Stony Brook University, Health Science Center, Stony Brook, New York, United States of America; ETH Zurich, Switzerland

## Abstract

The regulatory roles of sphingolipids in diverse cell functions have been characterized extensively. However, the dynamics and interactions among the different sphingolipid species are difficult to assess, because *de novo* biosynthesis, metabolic inter-conversions, and the retrieval of sphingolipids from membranes form a complex, highly regulated pathway system. Here we analyze the heat stress response of this system in the yeast *Saccharomyces cerevisiae* and demonstrate how the cell dynamically adjusts its enzyme profile so that it is appropriate for operation under stress conditions before changes in gene expression become effective. The analysis uses metabolic time series data, a complex mathematical model, and a custom-tailored optimization strategy. The results demonstrate that all enzyme activities rapidly increase in an immediate response to the elevated temperature. After just a few minutes, different functional clusters of enzymes follow distinct activity patterns. Interestingly, starting after about six minutes, both *de novo* biosynthesis and all exit routes from central sphingolipid metabolism become blocked, and the remaining metabolic activity consists entirely of an internal redistribution among different sphingoid base and ceramide pools. After about 30 minutes, heat stress is still in effect and the enzyme activity profile is still significantly changed. Importantly, however, the metabolites have regained concentrations that are essentially the same as those under optimal conditions.

## Author Summary

Sphingolipids play dual roles by serving as components of membrane rafts and by regulating numerous key cell functions. Although sphingolipids have been studied extensively, the details of their functioning are difficult to understand, because their synthesis, pathways of inter-conversion, and utilization constitute a complex, dynamically changing system. We analyze the role of yeast sphingolipids in the response to heat stress. Data show that the profile of these lipids changes almost immediately with the initiation of the stress, but it is *a priori* unclear how this response is organized. Using experimental data, a sophisticated dynamic model, and a novel optimization strategy, we show how changes in enzyme activities are temporally organized. Intriguingly, the results show that the cells take up as much material as possible in the first few minutes of heat stress and then shut down entry and exit routes of the biosynthetic pathway system. After about 30 minutes, when heat stress is still in effect, the enzyme activity profile is still significantly changed, but the metabolites have regained concentrations that are essentially the same as those under optimal conditions. The results demonstrate how novel insights are achievable with an effective combination of experimental and theoretical research.

## Introduction

Cells and organisms are regularly exposed to small fluctuations in their environments and have developed effective mechanisms of tolerance. Stronger perturbations lead to stresses, which are not as easily tolerated and require the cells to mount well-coordinated, multi-scale responses. These stress responses are very intriguing, because they offer superb windows into the complex strategies and mechanisms with which cells manage to live and thrive in a changing world. Heat is a particularly useful artificial stressor for microorganisms as it is easily applied and measured, and because cells and organisms have regularly experienced changes in temperature throughout evolution and developed very effective defenses.

It is well known that yeast reacts to modest heat stress with responses at several levels of its biological organization [1]–[3]. Numerous genes are up or down regulated within a few minutes, heat shock proteins are mobilized, transcription factors relocate between the cytosol and nucleus, the protective disaccharide trehalose begins to accumulate to high concentrations, and the metabolic profile of sphingolipids undergoes drastic changes. All these changes commence essentially immediately after a sufficient shift in temperature and may last for an hour or more. Some of these alterations, in turn, are known to serve as signals effecting secondary responses, for instance, by activating transcription factors and stress elements that trigger the expression of genes associated with heat stress.

An interesting aspect of the collective cellular responses is the fact that they occur at distinct time scales. Some are effective immediately, while others require involvement of the entire sequence of gene expression, transcription, translation and protein modification before the end result takes effect. We are slowly beginning to understand how these multi-scale responses are coordinated, but many details are still unclear. To gain further insight into the complexity of the response, Fonseca and collaborators recently presented strategies for designing mathematical models capable of capturing the multi-scale nature of heat stress responses in yeast [4], [5]. In particular, combining experimental information and computational techniques, these authors analyzed the trehalose heat stress system and demonstrated how it is organized at different biological levels and in different time domains.

In this article, we focus on the particular dynamic roles of sphingolipids in the heat stress response of the baker's yeast *Saccharomyces cerevisiae*. Specifically, we investigate how the cell establishes the observed alterations in sphingolipid profiles within a few minutes of heat stress. It is clear that these altered metabolite profiles are the result of changes in the activities of some or all enzymes of sphingolipid metabolism. We demonstrate that critical changes in activity can be inferred with a novel computational approach that uses measured times series of different sphingolipid concentrations, combined with a customized optimization strategy and a dynamic model that we have been developing and fine-tuning over the past decade [6]–[8].

### Heat Stress Responses and the Roles of Sphingolipids

Any substantial increase in temperature has a direct effect on the macromolecules in a cell. Among them, proteins and lipids are most strongly affected. Nucleic acids can denature upon exposure to heat, but this process requires much higher temperatures of about 75°C–100°C [9], which are outside the realm of tolerable heat stress.

Heat affects proteins in three ways. First, high temperature can modulate their synthesis from gene expression. In this context, Castells-Roca and colleagues investigated transcription rates and the stability of various mRNAs in *S. cerevisiae* following a temperature shift from 25°C to 37°C, and concluded that both were affected [10]. Second, processes of protein inactivation are temperature dependent. And third, heat can change a protein's folding state, which in turn may affect its function, as well as its removal by the proteasome. In particular, if the protein is an enzyme, its activity is influenced directly by its ambient temperature, according to an empirical relationship commonly called the Arrhenius effect or the Q_10_ effect.

Lipids are major constituents of membranes, and although the effects of heat are not completely understood, it appears that changes in temperature have an impact on membrane stiffness and fluidity [11]. Jenkins and coworkers [12] were among the first to connect sphingolipids to heat stress responses in yeast, demonstrating that these lipids play several particularly important roles (see also [13]–[17]). They subdivided the heat stress response into two phases. During the first phase, the cell needs to gain thermotolerance, which is at least partially accomplished with an accumulation of trehalose and the induction of heat shock proteins. Furthermore, the cell arrests its cell cycle in G0/G1, and this arrest lasts for approximately one hour, during which time there is no growth. Once thermotolerance is achieved, the cell culture starts growing again in the second phase of the response, even if the temperature is still elevated.

The first response phase is directly associated with two distinct features of sphingolipids. First, the structural characteristics of complex sphingolipids, together with sterols, contribute to the physical organization of specific membrane microdomains within membranes, called lipid-rafts. These rafts are known to be associated with membrane fluidity, protein compartmentalization, and protein sorting and trafficking through membranes (*e.g.*, [18]–[20]). As core components of rafts, sphingolipids are thus directly involved in organizational structures with potential signaling functions, and alterations in these functions are effective at a short time scale [21].

The second role of sphingolipids in the early heat stress response is their capacity to serve as bioactive signaling molecules. This signaling function influences the regulation of the cell cycle response, nutrient uptake, and the synthesis of proteins, which can have important secondary effects, especially if heat shock proteins are not available to serve as protectors of other proteins [22], [23]. Indeed, the groups of Ferguson-Yankey and Meier demonstrated that sphingolipid synthesis is required for an efficient initiation of translation, especially during heat stress [24], [25]. Specifically, the translation rate is increased if sphingoid bases are synthesized and accumulate. Jenkins and collaborators [26] and Dickson and co-workers [13] showed that ceramides and other simple sphingolipids, such as dihydrosphingosine and phytosphingosine (DHS and PHS), accumulate during heat stress in yeast. It appears that the short-term signaling role of sphingolipids is biphasic. In the first phase, sphingoid bases are required to regulate translation of heat shock mRNAs, a process that depends strongly on Pkh kinase, but not on Ypk kinases, which act downstream of Pkh. The second phase consists of a general increase in translation, which is dependent on the function of heat shock proteins. Without these heat shock proteins, the cell would run a severely elevated risk of protein aggregation or misfolding [25].

Sphingolipids also play roles over a longer time horizon. It has been known for a while that DHS induces the expression of a *STRE-LacZ* reporter gene, suggesting that the global stress element *STRE* can be activated by sphingolipid signals [13]. In particular, genes associated with the important trehalose stress response contain multiple copies of *STRE*. Knock-outs or overexpression of genes coding for the synthesis of dihydrosphingosine-1-phosphate (DHS-1P) show changes that resemble thermotolerant and heat sensitive yeast phenotypes, indicating that DHS-1P is an important regulator of heat stress [27]. Phytosphingosine-1-phosphate is involved with the regulation of genes required for mitochondrial respiration [28]. More generally, modulations in any of the sphingolipid enzymes cause ripple effects that change the concentrations of many sphingolipids and, possibly, the expression of a variety of genes. Futerman and Hannun [29] summarized the long-term signaling mechanisms of simple sphingolipids including sphingosine-1-phosphate, sphingosine, ceramide and ceramide-1-phosphate in yeast.

Taken together it is evident that sphingolipids exert important roles within the coordinated heat-stress responses of a cell, and that these roles are pertinent over short and long time horizons. However, it is so far unclear how the cell is able to establish an appropriate sphingolipid profile very quickly in response to heat stress. To answer this question, we propose a computational analysis based on observed heat stress time courses and a dynamic model of sphingolipid biosynthesis and degradation that allows us to investigate the dynamic profiles of critical enzymes involved in the sphingolipid pathway.

## Results

If changes in enzyme activities, for instance in response to heat, could be measured directly, the altered values could readily be entered into computational model equations [7], [8], and solving the equations would show the time trajectories of all pertinent metabolites. Our task here requires the opposite task, which is much more complicated. Namely, we ask: can we infer from the metabolite time courses which enzymes have to be altered dynamically, and by how much, in order for the model to generate the observed time-dependent metabolic profile? The optimization-simulation strategy proposed for this analysis, as detailed in the *Methods* section, answers this question and reveals for the first time how the activities of key sphingolipid enzymes are adjusted by the cell during the heat stress response. Specifically, we performed over 4,400 Monte-Carlo optimizations with random instantiations and selected from among these the 2,004 best models, based on the sum of squared errors (SSEs). To test the validity of these results, we also used the Akaike Information Criterion (AICc) for model selection and found that models selected based on SSEs and AICc were highly similar. Specifically, over 99% of the models identified through SSE were also identified with AICc; Text S1 contains further information on this comparative analysis. The thus selected models yielded dynamic trends for each sphingolipid enzyme during the heat stress response, These sets of individual trajectories reveal interesting insights. Namely, the trajectories collectively form tight, time-dependent activity ranges for those enzymes that control the influx to, and efflux from, the core of the sphingolipid biosynthetic pathway system. In other words, these enzymes always exhibit essentially the same dynamic activity patterns, independent of the randomly initialized start values. Most of the enzymes at the periphery of the pathway system, by contrast, exhibit widely varying activity profiles that are thus not identifiable from the available metabolic time series data. These results are described and discussed in detail in the following sections.

**Figure 1:**
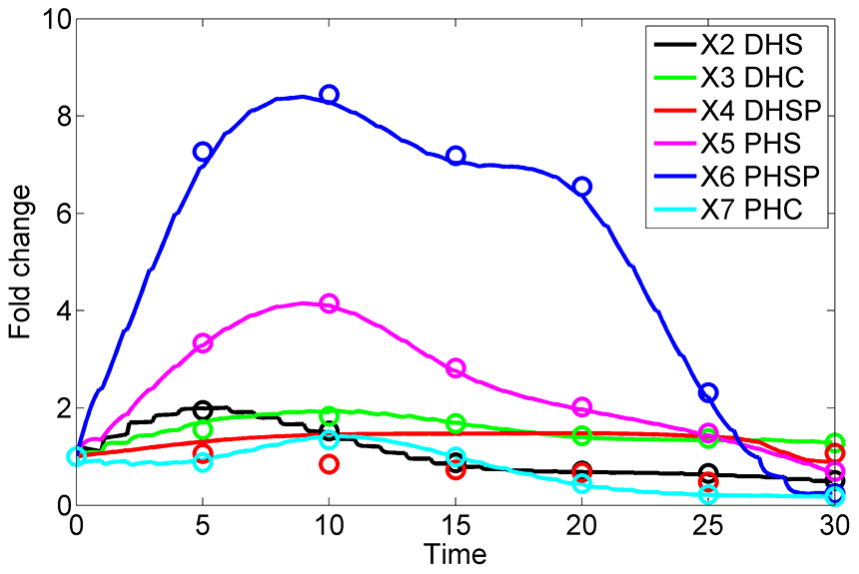
Data fit of the model with inferred enzyme activities. Using averaged trends in enzyme activities leads to simulated metabolic profiles (lines) that reflect the observations (symbols; averaged from two experiments) quite well. The lines are segmented, because the model is solved with enzyme activities that are constant from each time point to the next, when they are dynamically reset. The fold changes in DHS-P do not seem to be modeled very well. The reason is that the absolute concentration of this metabolite is very small (Figure 11) and any fold change becomes vastly amplified.

As a first validation of the collective results, we calculated the average of each computationally inferred enzyme activity at each time point and entered it into the pathway model (see *Methods* section) to check whether we were able to recoup the observed sphingolipid dynamics. The reconstructed sphingolipid dynamics indeed matches the original data quite well (Figure 1). This good match is by no means *a priori* guaranteed, because it is known that averages of parameter values from different good data fits do not necessarily correspond to good data fits themselves [30]. The averaged model was subsequently used for further interpretations of our results.

**Figure 2:**
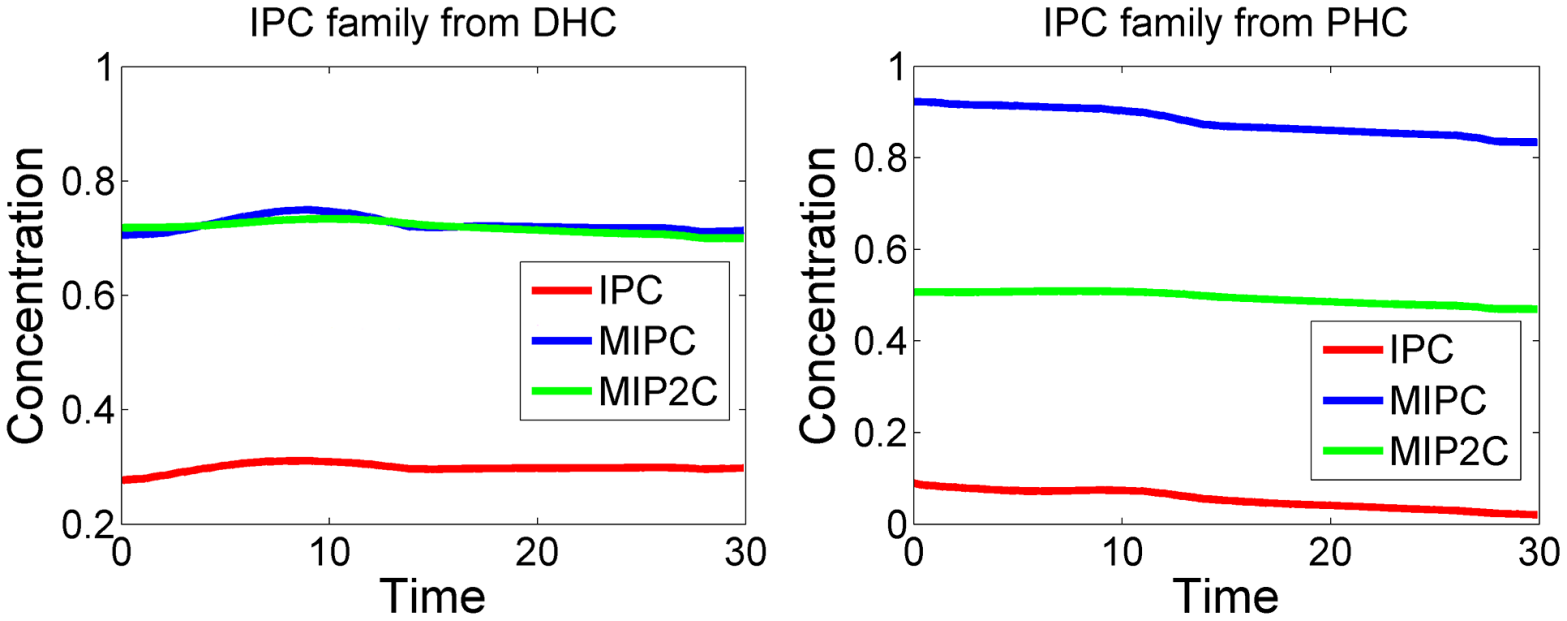
Concentration trends in complex sphingolipids. While the sphingoid bases and ceramides exhibit strong responses to heat stress (Figure 1), the complex sphingolipids IPC, MIPC, and M(IP)_2_C remain almost constant. The left and right panels show levels of members of the IPC family, derived from dihydroceramide and phytoceramide, respectively.

As a second, independent validation experiment, we explored changes in the concentrations of the complex sphingolipids IPC, MIPC, and M(IP)_2_C with the computationally inferred enzyme activities after a shift in temperature. In contrast to the profiles of simple sphingolipids (Figure 1), these trend lines are essentially flat, indicating that the complex sphingolipids do not change much during the heat stress response (Figure 2). This finding is directly consistent with experimental data [12] that were not used in our optimization.

**Figure 3:**
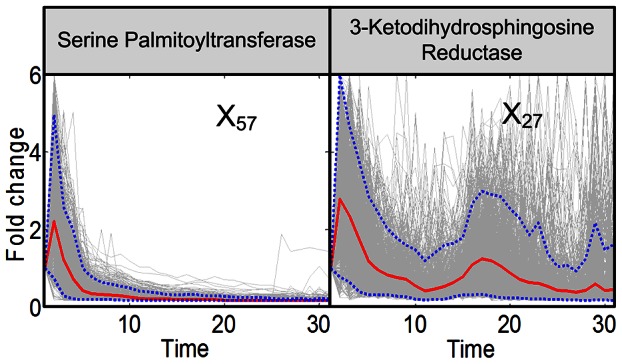
Trends in activities of enzyme at the entry point of sphingolipid biosynthesis. Serine palmitoyltransferase and 3-KDHS reductase are enzymes responsible for the production and degradation of 3-KDHS, which is the key initial metabolite of sphingolipid biosynthesis. The enzymes are located in the red zone of Figure 9. Grey lines are results of 2,000 individual iterations in the large-scale simulation. Red lines are ensemble averages, and dotted blue lines enclose 95% of the results.

**Figure 4:**
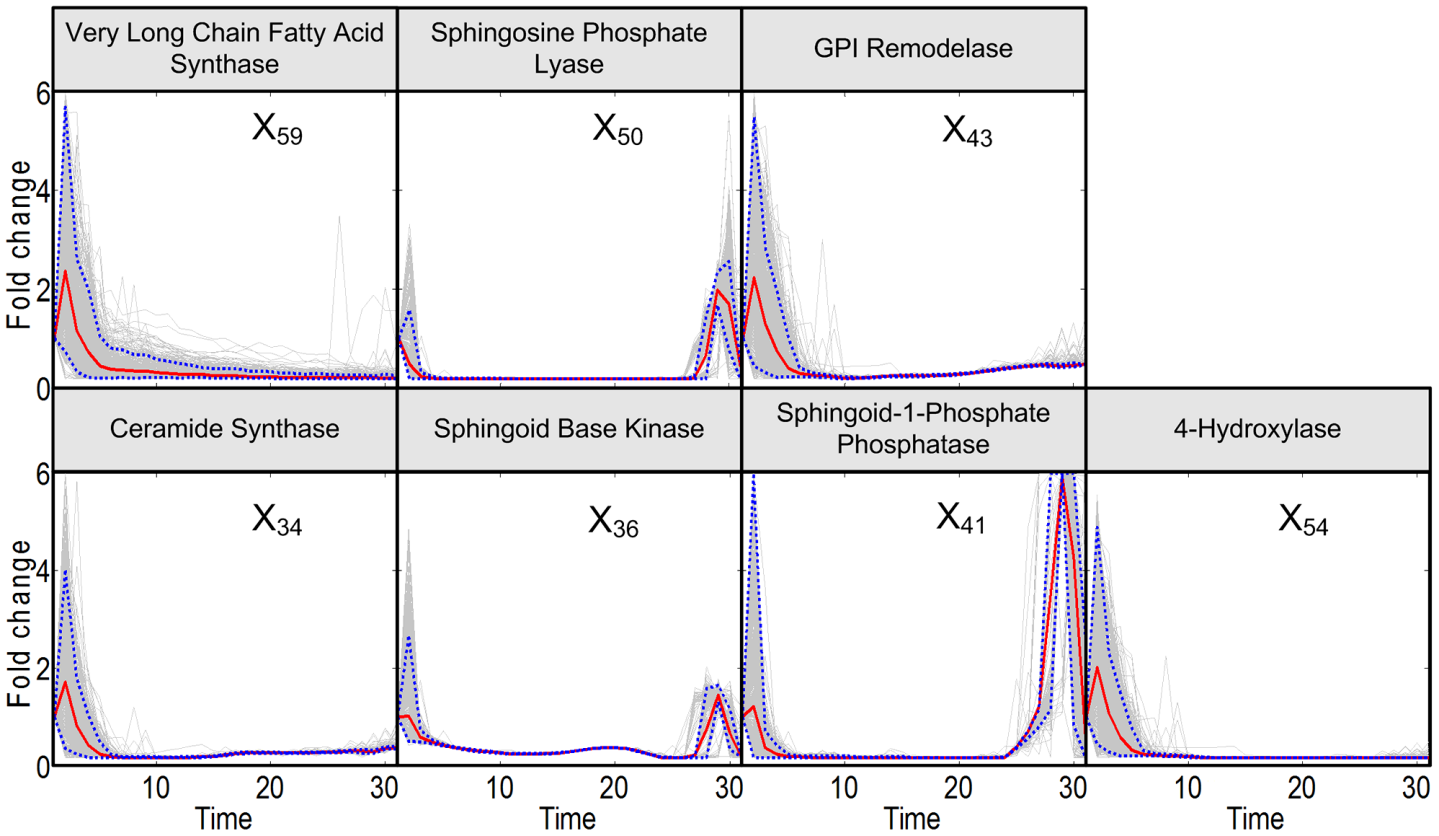
Trends in activities of enzymes in the core region of sphingolipid metabolism. After an initial spike, all enzyme activities in this region are reduced to almost nil. The enzymes are located in the blue zone of Figure 9. Grey lines are results of 2,000 individual iterations in the large-scale simulation. Red lines are ensemble averages, and dotted blue lines enclose 95% of the results.

As a final validation approach, and quasi as a negative control, we fixed those key enzymes that were inferred to have tight activity ranges (*X*_34_, *X*_36_, *X*_41_, *X*_43_, *X*_50_, *X*_54_, *X*_57_ and *X*_59_, see Figures 3 and 4) at their nominal steady-state values and optimized all other enzyme activity profiles with the same methods as before. The resulting fit (Figure S7) is not good and much inferior to that in Figure 1; further details regarding this negative control are given in Text S1.

**Figure 5:**
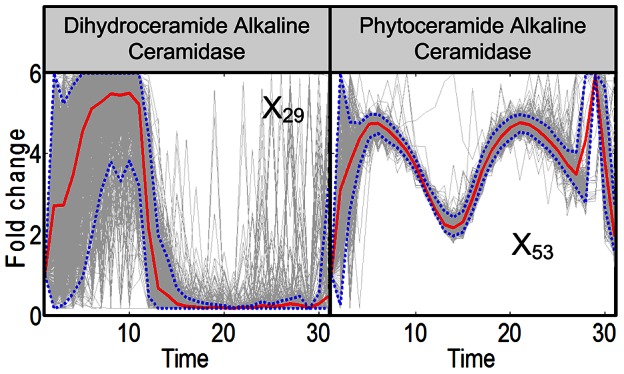
Trends in activities of the two alkaline ceramidases. Dihydroceramide alkaline ceramidase and phytoceramide alkaline ceramidase, which convert the ceramide form into sphingosines, exhibit distinct activity patterns. The enzymes are shown with light blue circles in Figure 9. Grey lines are results of 2,000 individual iterations in the large-scale simulation. Red lines are ensemble averages, and dotted blue lines enclose 95% of the results.

**Figure 6:**
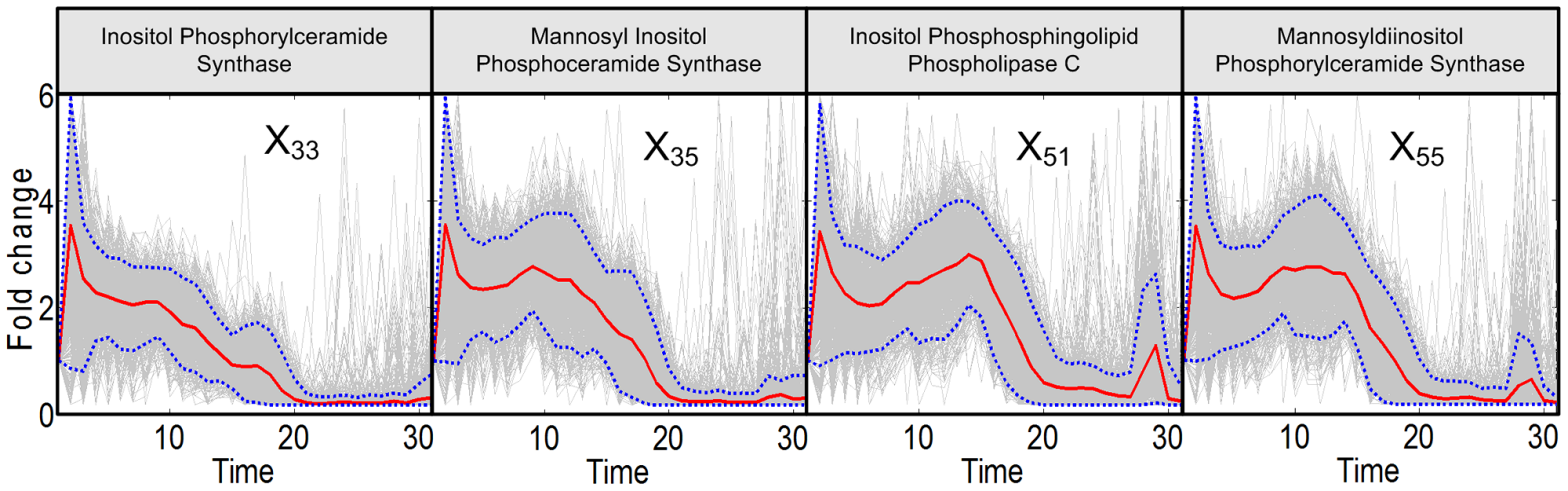
Trends in activities of enzymes associated with complex sphingolipids. Enzymes interconverting complex sphingolipids are at first hyper-active, but tend to lose most activity at some point between 20 and 30 minutes. The enzymes are located in the green zone of Figure 9. Grey lines are results of 2,000 individual iterations in the large-scale simulation. Red lines are ensemble averages, and dotted blue lines enclose 95% of the results.

**Figure 7:**
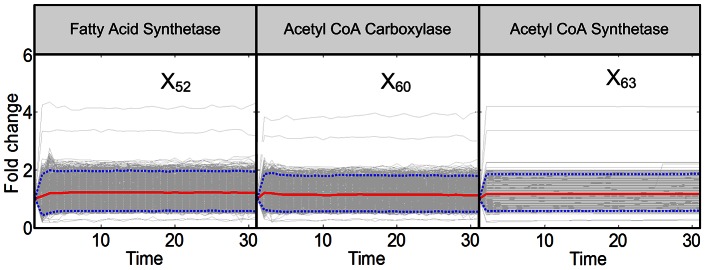
Trends in activities of enzymes associated with fatty acid CoA. The enzymes shown here are responsible for CoA enlongation. The enzymes are located in the yellow zone of Figure 9. Grey lines are results of 2,000 individual iterations in the large-scale simulation. Red lines are ensemble averages, and dotted blue lines enclose 95% of the results.

**Figure 8:**
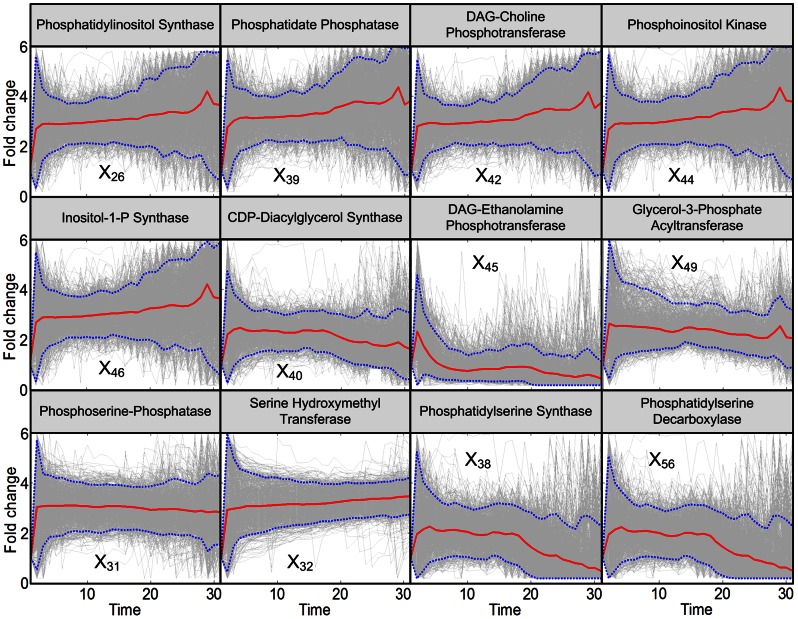
Trends in the remaining enzyme activities. Activities of enzymes at the periphery of the pathway system are not identifiable, mainly due to insufficient information and the fact that these enzymes are also involved in other pathways. Enzymes in the two upper panels are related to the phospholipid metabolism and enzymes in the lower panel are related to serine metabolism. The enzymes are located in the tan and pink zones of Figure 9. Grey lines are results of 2,000 individual iterations in the large-scale simulation. Red lines are averages, and dotted blue lines enclose 95% of the results.

**Figure 9:**
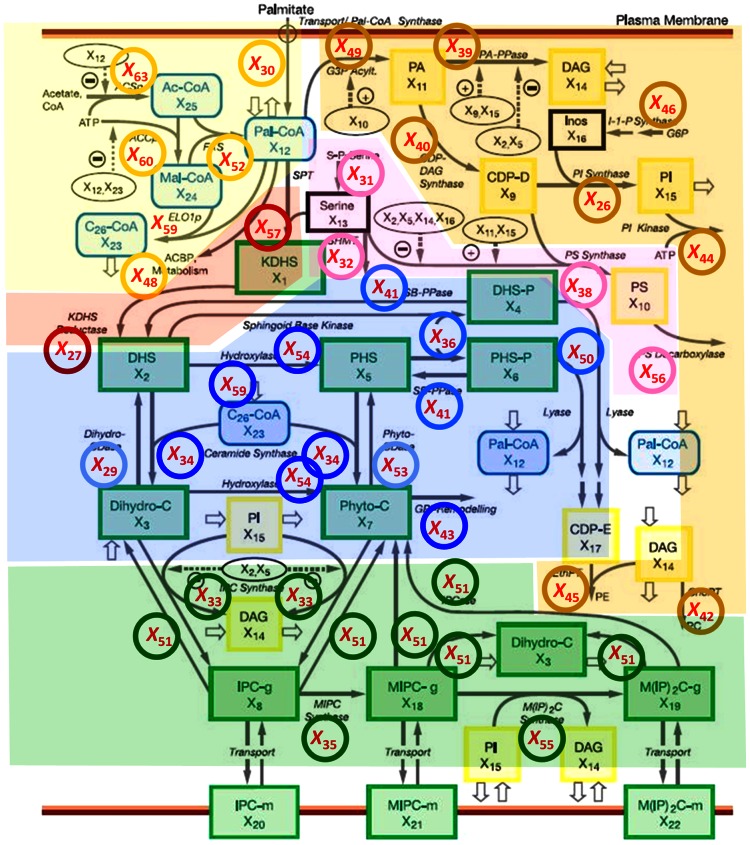
Zones of similar changes in enzyme activities. The zones correspond to enzyme profiles in Figures 3 (red), 4 and 5 (blue), 6 (green), 7 (yellow) and 8 (tan and pink) respectively. Abbreviations are: Green boxes (sphingolipid metabolism): KDHS (3 Ketodihydrosphingosine), DHS (Dihydrosphingosine), DHS-P (Dihydrosphingosine 1-phosphate), PHS (Phytosphingosine), PHS-P (Phytosphingosine 1-phosphate), DHC (Dihydroceramide), PHC (Phytoceramide), IPC-g (Inositol phosphorylceramide), MIPC-g (Mannosylinositol phosphorylceramide), M(IP)_2_C-g (Mannosyldiinositol phosphorylceramide), IPC-m (Plasma membrane inositol phosphorylceramide), MIPC-m (Plasma membrane mannosylinositol phosphorylceramide), M(IP)_2_C-m (Plasma membrane mannosyldiinositol phosphorylceramide). Yellow boxes (phospholipid metabolism): DAG (Sn-1,2-diacylglycerol), CDP-D (Cytidine diphosphate DAG), PS (Phosphatidylserine), PA (Phosphatidic acid), PI (Phosphatidylinositol), CDP-E (Cytidine diphosphate ethanolamine). Blue boxes (fatty-acid metabolism): Pal-CoA (Palmitoyl-Coenzyme), C26-CoA (Very long chain fatty acid), Mal-CoA (Malonyl coenzyme), Ac-CoA (Acetyl coenzyme). The base diagram was adapted from Alvarez-Vasquez *et al.*, *Nature* 433(7024): 425–430, 2005.

More interesting than these overall validation results are the trends in the individual enzyme activities (Figures 3–8). Each panel in each of these figures shows grey lines, which are often so dense that they seem to form shaded areas. Each line is one of 2,004 simulated trend lines and represents the computationally inferred activity of the given enzyme at time points 1, …, 30, given a random initialization at *t* = 0. The red line in each panel shows the mean of the trend lines, while the dotted blue lines enclose 95% of the grey trend lines. The collective results from these panels are visualized in a different manner in Figure 9, where they are superimposed on the sphingolipid pathway system.

The first enzyme of interest, serine palmitoyltransferase (SPT; *X*_57_) is the key bottleneck through which all *de novo* biosynthesis must pass (see red zone in Figure 9). The results show that the computationally inferred solution has SPT activity increasing briefly and then converging essentially to zero within a few minutes (Figure 3). This pattern is seen in essentially all 2004 simulations with random initial settings (see *Methods* Section. The representation of fold changes seems most intuitive. However, the same results are also presented in Text S1 on a log_2_ scale, which stretches reduced activity levels. Changes in the subsequent, very fast step (3KDHS reductase; *X*_27_) are less defined. A possible explanation is that the substrate of this reaction is toxic [31] and therefore never present in large concentrations, so that the capacity of the enzyme is not limiting. As a consequence, this enzyme activity does not contribute much to the error function that is to be minimized.

Similarly well defined as SPT are enzymes that catalyze the redistribution of material within the core of sphingolipid metabolism as well as the steps of sphingolipid removal (blue zone in Figure 9). These enzyme activities again rise quickly but approach a very small value shortly after (Figure 4). The very long chain fatty acid synthase and elongase (ELO1p; *X*_59_) is responsible for the delivery of fatty acid-CoA to the sphingolipid system, while sphingosine-phosphate lyase (*X*_50_) and GPI remodelase (*X*_43_) are the only true exit routes out of central sphingolipid metabolism. The remaining enzymes in this group redistribute material within the pathway. Ceramide synthase (*X*_34_) shows the same pattern as *X*_59_, *X*_50_, and *X*_43_, while sphingoid base kinase (*X*_36_), sphingoid-1-phosphate phosphatase (*X*_41_), and 4-hydroxylase (*X*_54_) exhibit the same initial phase, but begin to rise more or less strongly after about 25 to 28 minutes of heat stress. These late increases in activity apparently indicate the first consequence of heat-induced gene expression. Among these enzymes, sphingoid-1-phosphate phosphatase (*X*_41_) shows the strongest peak at 28 to 30 minutes by far. This enzyme is known to be a key regulator of sphingolipid metabolism and, in particular, of stress responses [15]. It plays an important role in regulating the crucial balance between ceramide and phosphorylated sphingoid base levels and thereby modulates later stress responses.

The two alkaline ceramidases exhibit rather different patterns. As with the previous enzymes, the activity of dihydroceramide alkaline ceramidase (dihydro-CDase; *X*_29_), which converts dihydroceramide into dihydrosphingosine, decreases to almost zero, but much later and in a less defined manner. By contrast, the activity of phytoceramide alkaline ceramidase (Phyto-CDase; *X*_53_) shows tight trends consisting of three peaks, before returning to normalcy after about 30 minutes (Figure 5). These differences indicate that there is no “symmetry” between the function of dihydro- and phyto-forms of sphingolipids.

The activity patterns of enzymes associated with complex sphingolipids are different; they are shown in Figure 6 (green zone in Figure 9). They all indicate a sustained level of hyper-activity for about 20 minutes, before becoming very low between about 20 and 28 minutes. These enzymes are inositol phosphorylceramide synthase (IPC synthase; *X*_33_), mannosyl inositol phosphoceramide synthase (MIPC synthase; *X*_35_), and mannosyl di-inositol phosphorylceramide synthase (M(IP)_2_C synthase; *X*_55_), as well as inositol phosphosphingolipid phospholipase C (IPCase; ISC1 *X*_51_), which returns IPC, MIPC and M(IP)_2_C to the dihydroceramide (DHC) and phytoceramide (PHC) pools.

The remaining enzyme activities are not identifiable with our analysis. Some appear to be essentially unchanged throughout the measurement period of 30 minutes, during which the temperature remains elevated. Examples are fatty acid synthase (*X*_52_), acetyl-coenzyme A carboxylase (*X*_60_), and synthase (*X*_63_) (Figure 7; yellow zone in Figure 9). Other enzyme activity patterns (*X*_26_, *X*_39_, *X*_42_, *X*_44_, *X*_46_, *X*_40_, *X*_45_, *X*_49_, *X*_31_, *X*_32_, *X*_38_, and *X*_56_) exhibit larger degrees of variation (Figure 8; pink and tan zones in Figure 9). On average, each pattern exhibits an individual Q_10_ effect, and subsequently stays more or less constant, decreases somewhat, or continues to increase slightly, but the trends are not clear. One reason for the large variability in these trends may be that the available metabolite data are not informative enough. It is also to be expected that the different processes catalyzed by these enzymes allow for a large degree of redundancy. For instance, serine is not only used in the SPT reaction, but also for the production of phosphoserine and in the serine hydroxymethyl transferase reaction, so that computationally inferred excesses in one reaction may be compensated numerically by a lower activity of one of the other two. Finally, as we discussed elsewhere [7], it is possible that these processes are not as well modeled as those at the core of sphingolipid biosynthesis, because they also participate in other pathway systems, such as phsopholipid or ergosterol metabolism.

The computationally inferred patterns in enzyme activities are collectively depicted as colored zones in Figure 9. Most interesting are the red and blue zones, which control the influx to, redistribution within, and efflux out of the core of sphingolipid bipsynthesis. The green zone contains the complex sphingolipds, which provide material for activities in the blue zone, even though their concentrations do not change much throughout the thirty minutes of heat stress (see Figure 2). The yellow, pink, and tan zones at the periphery contain fatty-acid CoAs, serine compounds and phospholipids, respectively. These are necessary for sphingolipid biosynthesis, but also for other pathways. Due to their multiple roles, they are presumably not modeled comprehensively, and their enzyme activities are not identifiable with the data and methods used here.

The trend lines, as well as their averages, collectively suggest that the sphingolipid heat stress response is achieved with quite moderate changes in many enzymes rather than very extensive changes in just a few key enzymes. This result is consistent with earlier studies in the context of the diauxic shift, which implied that cells probably satisfy altered metabolic demands with many small, rather than a few large, adjustments [6], [32]. While it is impossible to identify the true advantage of this strategy unambiguously, the avoidance of large changes in any of the system components might be expected to reduce the risk of undesired side effects in neighboring pathways.

All trends in enzyme activities follow distinct patterns, which are the results of a balance among three forces induced by the shift in temperature from 30°C to 39°C: first, an essentially immediate increase in activity to a level of up to about four times the baseline, according to the enzyme's (typically unknown) Q_10_ value, which quantifies the Arrhenius effect (see Table S3); second, a possibly diminished activity due to partial protein unfolding and/or an altered half-life of the corresponding protein and/or mRNA; and third, changes in enzyme activity due to regulation and/or gene expression. These forces may be active to different degrees in overlapping time windows.

The three forces lead to different activity patterns. Most striking is the set of enzymes controlling the influxes and effluxes associated with the core of sphingolipid biosynthesis. Their pattern of heat responses consists of enzyme activities that first exhibit a Q_10_ effect, which is subsequently counteracted by deactivation mechanisms that could be due to changes in RNA amounts, changes in half-lives or degradation rates of proteins or mRNAs, post-translational modifications, or heat induced gene depression [33]. Thus, after a few minutes, these enzyme activities essentially disappear.

### Overall Heat Stress Response Strategy

**Figure 10:**
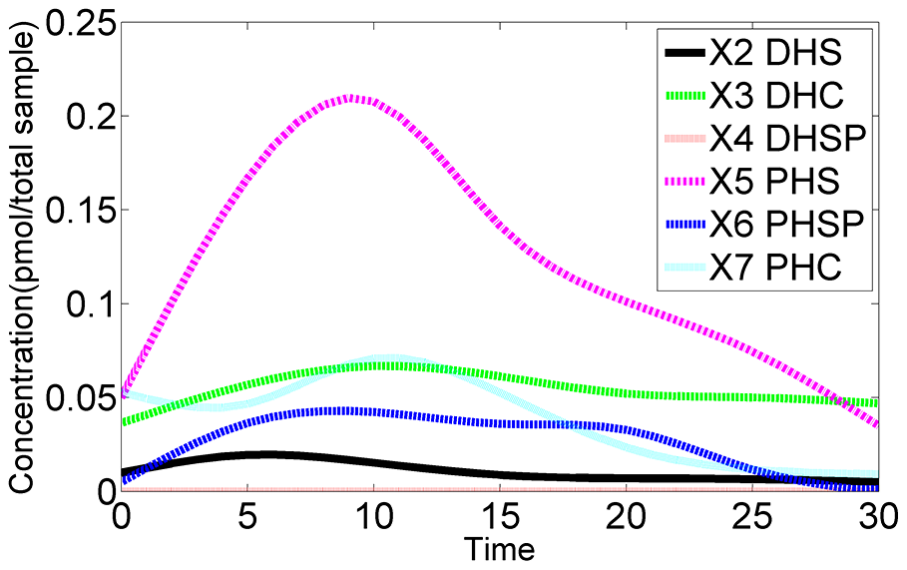
Smoothed time series data. Absolute changes in six key sphingolipid metabolites in response to a temperature shift from 30°C to 39°C at time 0. The raw data were smoothed with a standard spline technique. See also Figure 1.

Without any computational analysis, the measured data directly show which sphingolipids are apparently needed under heat stress at different points in time. Measured as absolute quantities, PHS increases by far the most in concentration, whereas PHS-P increases most relative to its baseline value. Interestingly, both adjustments are much stronger than in the corresponding dihydro-forms. For instance, the concentration of DHS-P remains very low throughout the observation period of 30 minutes (Figure 10). DHS reaches its modest peak earlier than PHS and PHS-P, whereas PHC reaches its peak later. It is difficult to discern the rationale for this timing and the differences in peak heights.

**Figure 11:**
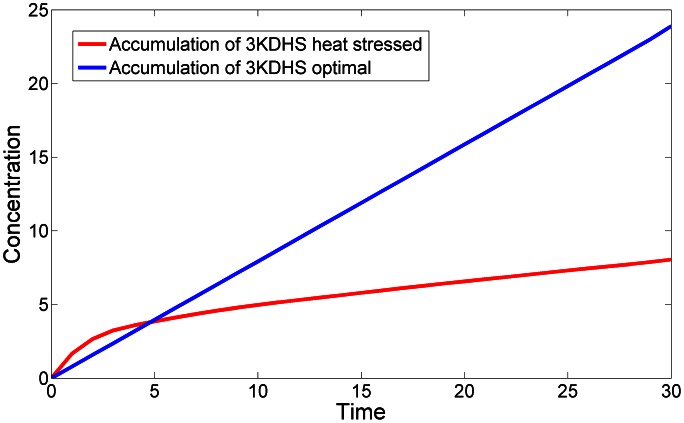
Changes in 3KDHS production under optimal and heat stress conditions. The model allows the computation of 3KDHS accumulation without 3KDHS degradation. The accumulation patterns are distinctly different under optimal (blue) and heat stress (red) conditions.

What the computational analysis shown here suggests is how these observed adjustments are implemented by the cell. Initially, *de novo* biosynthesis increases quickly, but only for the first three or four minutes. The model actually allows us to quantify and compare the total amount of biosynthesis under optimal and heat stress conditions. Namely, we can record in the dynamic simulation the total production of 3-KDHS, while computationally omitting its degradation (Figure 11). Under optimal conditions, and with a constant influx of palmitate and serine, this accumulation is linear (blue line), and considering consumption as well, the concentration of 3KDHS is constant (results not shown). By contrast, under heat stress, the accumulation is faster for the first few minutes (red line), but it is increasingly reduced subsequently. Considering consumption as well, the concentration of 3KDHS decreases (results not shown).

In the next five to ten minutes, the patterns diverge strikingly. Probably most intriguing, both the input to, and the exit from, central sphingolipid metabolism are almost completely shut down. During this time period, the cell not only counteracts the unavoidable Q_10_ effect in SPT, but down-regulates this enzyme to a mere residual amount, as shown in top left panel of Figure 3. Similarly, the exit routes through the lyase and remodelase steps lose activity about 5 minutes into the heat stress (Figure 3). The second step of *de novo* biosynthesis, KDHS reductase, is less dramatically affected (right panel in Figure 3), but deprived of substrate. This substrate deprivation appears to be safer than enzyme down-regulation, as 3KDHS is toxic [34] and any accumulation could be dangerous.

The computational deductions imply that *de novo* sphingolipid biosynthesis appears to be up-regulated only for the first few minutes [12]. To establish the needed changes in sphingolipid profile under heat stress, the cell appears to absorb and process residual substrate as vigorously as possible, but subsequently seems to count on the much more reliable use of existing complex sphingolipids for the generation of signaling molecules such as PHS, PHS-P and, to a lesser degree, DHS and DHC, and on a subsequent redistribution among the simple sphingolipid pools. This conclusion is based on the inferred reduction in biosynthesis after about five minutes, the shutting off of the lyase and remodelase steps, as well as three additional observations. First, IPCase (Figure 6) is strongly upregulated in a sustained manner for about 15 minutes. Second, the hydroxylase, which converts DHC into PHC and DHS into PHS, loses almost all activity throughout the measured time period (Figure 4). Third, processes leading to the synthesis of complex sphingolipids, including IPC synthase and the synthesis of PI and DAG, are down-regulated after about 15 minutes (Figure 6), thereby slowing down the genesis of new complex sphingolipids from simple sphingolipids. Several of the enzymes associated with complex sphingolipids begin to become active again about 28 minutes into the heat stress, which may be a consequence of changes in gene expression.

After 30 minutes, the six measured sphingolipid concentrations essentially return to their baseline levels. In stark contrast, the enzyme system has not returned to its original state, and several enzymes still exhibit an activity that is quite distinct from the profile under optimal temperature conditions. Thus, the cell, which is still under heat stress, is regaining a close resemblance of normalcy with respect to its metabolites, but this state is achieved with a significantly different flux and enzyme profile.

## Discussion

In this work, we have proposed a computational approach to analyze heat stress response strategies in yeast. Specifically, we have inferred how cells adjust their enzyme activities within sphingolipid metabolism, which has been demonstrated in numerous earlier reports as a heat sensitive signaling system. Using experimental measurements of metabolite concentrations following a shift in temperature, combined with a detailed dynamical model, we computationally inferred adjustments in enzyme activities that appear to be both sufficient and necessary for mounting the observed metabolic response. Rather than computing a single solution to the inverse task, we computed a comprehensive ensemble of over 4400 independent solutions and selected from among them the best 2004 solutions, based on SSE and AICc metrics. These 2004 solutions led to very similar trends in the activities of key enzymes, although not of enzymes at the periphery of the pathway system.

The computed results suggest, first, that the response to heat is not achieved by drastic changes in a few “key” enzymes, but that numerous enzymes are involved. Second, the dynamic alterations in activities differ substantially in both, magnitude and timing, as well as in the general shape of the enzyme activity trends throughout the observed 30-minute time window following the initiation of heat stress. The main surprise in our results is the deduction that the changes in sphingolipid profile are apparently not achieved by sustained increases in *de novo* biosynthesis but through a brief initial spike, followed by the retrieval of simple sphingolipids from membrane-associated complex sphingolipids, as well as a complicated redistribution scheme among the different ceramide and sphingosine forms. While this strategy was not expected, its seems to have merit, because the cell cannot be sure that new resources are quickly available for *de novo* synthesis of sphingolipids, while complex sphingolipids such as IPC, MIPC and M(IP)_2_C are integral components of membranes and therefore always available, with the possible exception of the most deprived situations. Thus, it seems that the cell sacrifices some of its membrane structures and recreates them once the stress situation is under control. This sacrifice, however, is not very substantial, as the concentrations of complex sphingolipids change very little during the heat stress response (Figure 2). These results are consistent with experimental finding of Jenkins *et al.* [12], who studied different roles of sphingolipids during the heat stress response. Using isotope labeling, they showed that sphingoid bases and ceramides increase early on via *de novo* synthesis, but that IPC, MIPC and M(IP)_2_C remain essentially constant over a period of more than one hour. Wells *et al.* [17] also studied the formation of ceramide in response to heat stress and, using labeled phosphosphingolipids, and concluded that ceramide formation from IPC, MIPC, and M(IP)_2_C through the IPCase reaction was unlikely. However, the concentration profiles these authors observed were very different from those obtained by Cowart et al. [28], which we used here. In particular, under Wells' 39°C treatment, ceramide remained elevated at a level five times its baseline throughout the two-hour measurement period. Outside the fact that these authors studied a temperature shift from 24°C to 39°C, the differences in concentration profiles to those used here (Figure 1; Cowart et al. [28]) remain unexplained.

Although the computational results were obtained without any particular assumptions, some uncertainties are associated with the fact that many of the intermediate sphingolipids had not been measured and that the mathematical approach may not have revealed the one truly optimal solution. For instance, all results are obtained from large-scale simulations with a dynamical model that has been validated to some degree but could certainly be improved. Given the present data, it is unlikely that further simulations of the same type as shown here would lead to different results. However, if other metabolite concentrations could be measured, or if it were possible to determine some internal metabolic fluxes independently of the metabolite concentrations, the degree of reliability of our results would greatly increase.

The study presented here elucidates a systemic strategy with which the cell establishes the observed sphingolipid profile, but it does not address the specific roles of the various sphingolipids in the heat stress response. Interestingly, some of the simple sphingolipids that are known to have signaling roles do not change all that much, while others do. In particular, DHS, which activates the stress element STRE in the expression of stress related genes, maximally rises to only about twice its normal level, about 5 minutes into the heat stress. Apparently, this increase is sufficient. By contrast, PHS-P, which was recently identified as an important gene regulator, rises to a level that corresponds to almost 10 times its baseline level and exhibits a sustained response that lasts over 20 minutes. PHS rises to a four-fold level. No direct signaling role is known, and it may just be that this compound is needed as a precursor of PHS-P.

The experiments generating the data used here exposed the cells to persistent heat stress. At the end of the 30-minute observation period, all six key sphingolipids have essentially returned to their normal levels, except for DHC, which still seems to be very slightly elevated. By contrast, many of the enzyme activities are not “back to normal.” Expressed differently, the cell manages to mount a strong transient response, which is known to lead to longer-term genomic responses. Subsequently, within a total of just 30 minutes, it is able to adjust its catalytic machinery to the persistent heat conditions in such a manner that the fluxes exhibit a distinctly different activity pattern which, nevertheless, re-establishes a favorable metabolic state that is remarkably close to that under optimal conditions.

Our focus on sphingolipids sheds light on just one aspect of the well-coordinated, complex responses with which yeast adjusts to a new environmental condition. Nonetheless, this particular aspect is of special interest, as the roles of sphingolipids and their biosynthetic pathways have been preserved throughout evolution, from yeast to humans, where they are involved in numerous differentiation and disease processes (*e.g.*, [35]–[38]).

## Methods

### Data

The data, previously obtained in one of our labs, were described in the literature (see Supplements of [28]). They consist of duplicate 30-minute time courses of six key sphingolipids, collected following a step increase in temperature from 30°C to 39°C. Specifically, changes in metabolite concentrations were measured at baseline (*t* = 0; normal temperature) and at 5, 10, 15, 20, 25, and 30 minutes of heat stress. We used these measurements, averaged the duplicates, and then applied a smoothing spline technique to interpolate the trend of each time course so that concentration values at 31 time points (0, 1, …, 30 minutes) became available for each sphingolipid. The smoothed transients are shown as absolute concentrations in Figure 11 (see also Figure 1 for fold changes, which shows the smoothed data as symbols, along with a model fit based on averaged enzyme activities). For our computational analysis we used relative changes in each sphingolipid with respect to the baseline steady state before heat stress, which we directly obtained from the time series measurements, and scaled these with steady-state values, which were described in earlier work [8], to obtain actual concentrations.

### Mathematical Model

The biosynthesis, metabolic conversions, and degradation of sphingolipids constitute a complex, highly regulated pathway system (Figure 9) that exceeds intuitive capabilities and suggests computational modeling for quantitative systemic analyses. Over the past decade, we have developed a series of such models using a General Mass Action (GMA) formulation within the modeling framework of Biochemical Systems Theory (BST) [6]–[8], [39]. Because these models have been described in detail elsewhere, we can keep their description here to a minimum.

The simple and complex sphingolipids, as well as other pertinent metabolites, are represented in the model as dependent variables, each of which satisfies an ordinary differential equation (ODE). Each ODE contains representations of the processes that produce or degrade this metabolite. According to the tenets of BST, each process is represented as a product of power-law functions, which consists of a rate constant and of every variable directly affecting this process, raised to an exponent, called a kinetic order. Variable names and equations are presented in Text S1 and an SBML implementation can be found in the file Model S1.

As an example for how to design a system equation, consider the dependent variable 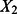, which represents dihydrosphingosine (DHS). This metabolite is generated from three possible sources. First, KDHS reductase (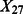) catalyzes the reduction of 3-keto-dihydrosphingosine (KDHS; 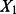). The formulation of this process consists of a rate constant 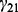, which is multiplied by 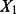, raised to the kinetic order 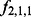, and by 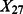, raised to the kinetic order 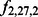. Thus, the reduction process is modeled as 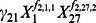. Second, DHS can be produced from dihydrosphingosine-1-phosphate (DHS-P; 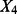), a process catalyzed by sphingoid 1-phosphate phosphatase (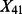). In analogy to the first process, this step is represented with its own rate constant, as well as the substrate and enzyme, which are both raised to appropriate kinetic orders. Third, dihydroceramide alkaline ceramidase (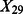) converts dihydroceramide (DHC; 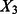) into DHS, and this process is formulated in an analogous manner. DHS is subject to three possible metabolic fates, namely through the ceramide synthase reaction toward DHC, through the 4-hydroxylase reaction toward phytosphingosine (PHS), and through the sphingoid base kinase reaction toward DHS-P. Taken together, the ODE equation describing the dynamics of DHS contains three influx terms and three efflux terms as shown in Eq. (1).
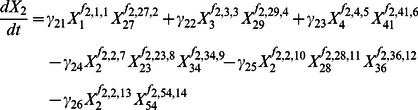
(1)All differential equations for dependent variables are formulated in this manner. Values for all parameters were determined from the literature [8], [40]. The complete model consists of 25 ordinary differential equations, including those representing the six key sphingolipids of interest here, namely dihydrosphingosine, dihydroceramide, dihydrosphingosine 1-phosphate, phytosphingosine, phytosphingosine 1-phosphate and phytoceramide. The model furthermore contains 41 independent variables, which represent enzyme activities and metabolites such as ATP, palmitate, acetate and phosphoserine, which were assumed to be constant or considered unaffected by the dynamics of the pathway system. The model was rigorously tested and validated against data not used for model construction [7]. It was also recently combined with a model of the sterol pathway, which has relevance for the composition of membrane rafts [39]. An SBML version of the model can be found in zip file Model S1.

### Piecewise Optimization Approach

As stated at the beginning of the *Results* section, it is our task to infer from the measured metabolite time courses which enzymes have to be altered dynamically, and by how much, in order for the model to generate the observed time-dependent metabolic profile? Mathematically, this inverse problem is underdetermined and furthermore complicated by the fact that the pathway is described by a system of nonlinear differential equations, as discussed before. If we were only concerned with a baseline steady state and the move of the system to a new steady state appropriate for heat stress conditions, we could use methods of linear algebra and pseudo-inverses, as we have demonstrated elsewhere [32]. However, here we are interested in the entire trajectories between stimulus (*i.e.*, the beginning of heat stress) and the cell's metabolic adjustments over 30 minutes.

We solved this dynamic inverse problem with an iterative, piecewise optimization approach. Specifically, we estimated optimal enzymatic profiles by minimizing the distance between the smoothed sphingolipid data and the simulation results at each time point, with 1-minute time intervals, from 0 to 30 minutes. At each time point, the optimization engine searched for the best set of enzyme activities, which were modeled as independent variables. To satisfy the specified objective function, we algorithmically minimized the distances between the six observed sphingolipid concentrations and the solutions produced by each trial set of independent variables. We executed this strategy 4144 times, using different random values for initial settings. We then selected the 2004 best models based on residual errors (SSEs). In order to test the performance of this metric, we also selected models based on the Akaike criterion (AICc), and both criteria produced very similar results. Please see Text S1 for a detailed comparison of results using these two criteria. Subsequently, scanning all solutions throughout the 30-minute time period yielded dynamic alteration profiles in all enzymes as well as corresponding metabolite profiles that were consistent with the observed profiles throughout the experimental time period. Further details of this procedure are presented in the Text S1.

Each optimization run produced a dynamic enzymatic profile throughout the time period from 0 to 30 minutes. Due to the randomization of initial values and to the fact that the system is underdetermined, the solutions from different runs were different. Thus, instead of searching for a single unique solution, we studied an entire large ensemble of solutions and asked whether the solutions would reveal consistent trends of enzymatic profiles with in the potentially large solution space. Indeed, the overall result of this strategy was a set of surprisingly tight ranges for the key enzymes of sphingolipid biosynthesis.

## Supporting Information

Figure S1 **Trends in activities of enzyme at the entry point of sphingolipid biosynthesis.** Serine palmitoyltransferase and 3-KDHS reductase are enzymes responsible for the production and degradation of 3-KDHS, which is the key initial metabolite of sphingolipid biosynthesis. Grey lines are results of 2,000 individual iterations in the large-scale simulation. Red lines are ensemble averages, and dotted blue lines enclose 95% of the results. The figure corresponds to Figure 3 of the main text.
(TIF)

Figure S2 **Trends in activities of enzymes in the core region of sphingolipid metabolism.** After an initial spike, all enzyme activities in this region are reduced to almost nil. Grey lines are results of 2,000 individual iterations in the large-scale simulation. Red lines are ensemble averages, and dotted blue lines enclose 95% of the results. The figure corresponds to Figure 4 of the main text.
(TIF)

Figure S3 **Trends in activities of the two alkaline ceramidases.** Dihydroceramide alkaline ceramidase and phytoceramide alkaline ceramidase, which convert the ceramide form into sphingosines, exhibit distinct activity patterns. Grey lines are results of 2,000 individual iterations in the large-scale simulation. Red lines are ensemble averages, and dotted blue lines enclose 95% of the results. The figure corresponds to Figure 5 of the main text.
(TIF)

Figure S4 **Trends in activities of enzymes associated with complex sphingolipids.** Enzymes interconverting complex sphingolipids are at first hyper-active, but tend to lose most activity between 20 and 30 minutes. Grey lines are results of 2,000 individual iterations in the large-scale simulation. Red lines are ensemble averages, and dotted blue lines enclose 95% of the results. The figure corresponds to Figure 6 of the main text.
(TIF)

Figure S5 **Trends in activities of enzymes associated with fatty acid CoA.** The enzymes shown here are responsible for CoA elongation. Grey lines are results of 2,000 individual iterations in the large-scale simulation. Red lines are ensemble averages, and dotted blue lines enclose 95% of the results. The figure corresponds to Figure 7 of the main text.
(TIF)

Figure S6 **Trends in the remaining enzyme activities.** Activities of enzymes at the periphery of the pathway system are not identifiable, mainly due to insufficient information and the fact that these enzymes are also involved in other pathways. Enzymes in two upper panels are related to the phospholipid metabolism and enzymes in the lower panel are related to serine metabolism. Grey lines are results of 2,000 individual iterations in the large-scale simulation. Red lines are averages, and dotted blue lines enclose 95% of the results. The figure corresponds to Figure 8 of the main text.
(TIF)

Figure S7 **A negative control experiment.** When the key enzymes are locked into their normal activity values and all other enzyme activities are allowed to be optimized, the fit of the best model to the experimental data is not very good.
(TIF)

Figure S8 **Sums of squared errors for individual optimizations.** Upper panel: SSEs for 2,000 simulations with the original model. Lower panel: SSEs for 200 simulations with the constrained model. The *X*-axis shows the index of each individual simulation, while the *Y*-axis shows the corresponding sum of squared errors (SSE); note different scales.
(TIF)

Figure S9 **Distributions of sums of squared errors for individual simulations.** The distribution on the left contains SSEs for the model in which all enzymes are allowed to change. The distribution on the right contains the corresponding SSE values for the constrained model.
(TIF)

Figure S10 **Comparison of data fits.** Left panel: Data fitted with the unconstrained averaged model (identical to Figure 1 of the text). Right panel: 179 data fits with individual model simulations that resulted in SSE < 1.25×10^−5^ (*cf.* Figure S8).
(TIF)

Figure S11 **The histogram of AICc values of the 4144 initial models clearly indicates that the 2018 models in the left-most column are superior to all other parameterizations.** 99.35% (1991) of the 2004 models identified by SSE fall into this column, thereby demonstrating very strong consistency between the two measures of quality.
(TIF)

Table S1 **Metabolites, enzymes, abbreviations, and variable names.**
(DOCX)

Table S2 **Summary of identifiable dynamic changes in enzyme activities in response to heat stress.**
(DOCX)

Table S3 **Estimated Q_10_ values, based on the initial increases in enzyme activities.**
(DOCX)

Model S1 This file contains different components of an implementation of the base model in SBML format.
(ZIP)

Text S1 This file contains model equations, model details in Tables S1, S2, S3, details regarding the optimization procedure, an alternative representation (Log_2_) of trends in enzyme activities, an estimation of Q_10_ values for enzymes of the sphingolipid pathway, an assessment of simulation results with a “negative control,” comments on the Akaike Information Criterion, and additional references.
(DOCX)
