# Supplementary figures and images for: Coordination of Rapid Sphingolipid Responses to Heat Stress in Yeast
Source: PLoS Comput Biol. 2013 May 30;9(5):e1003078. doi: 10.1371/journal.pcbi.1003078 (PMC3667767; doi:10.1371/journal.pcbi.1003078)

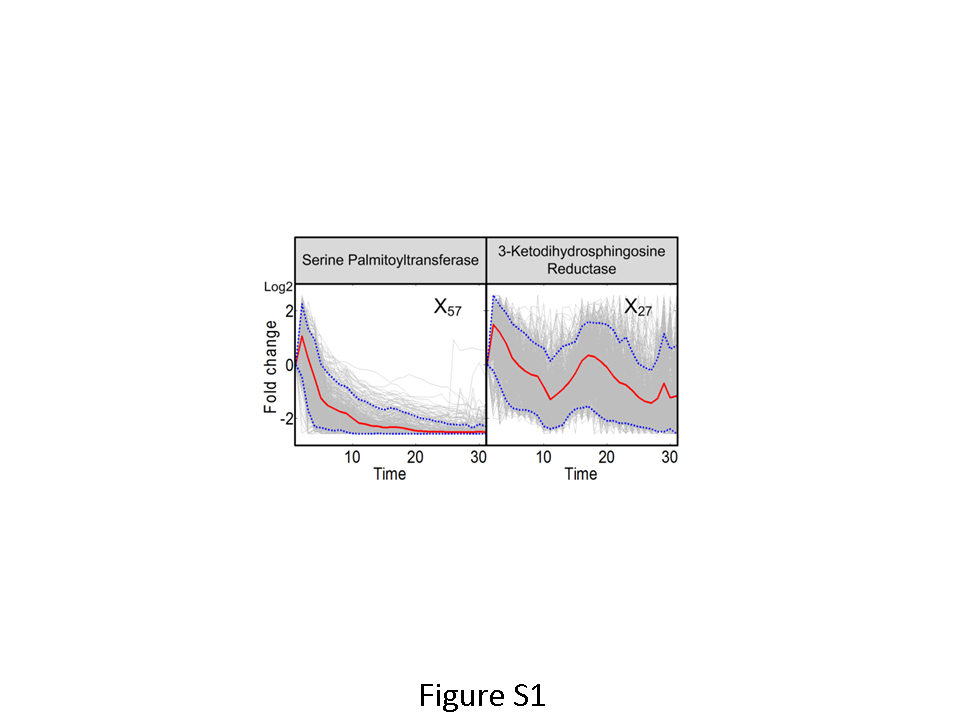

Supplement: Figure S1 — Trends in activities of enzyme at the entry point of sphingolipid biosynthesis. Serine palmitoyltransferase and 3-KDHS reductase are enzymes responsible for the production and degradation of 3-KDHS, which is the key initial metabolite of sphingolipid biosynthesis. Grey lines are results of 2,000 individual iterations in the large-scale simulation. Red lines are ensemble averages, and dotted blue lines enclose 95% of the results. The figure corresponds to Figure 3 of the main text. (TIF) [file pcbi.1003078.s001.tif]

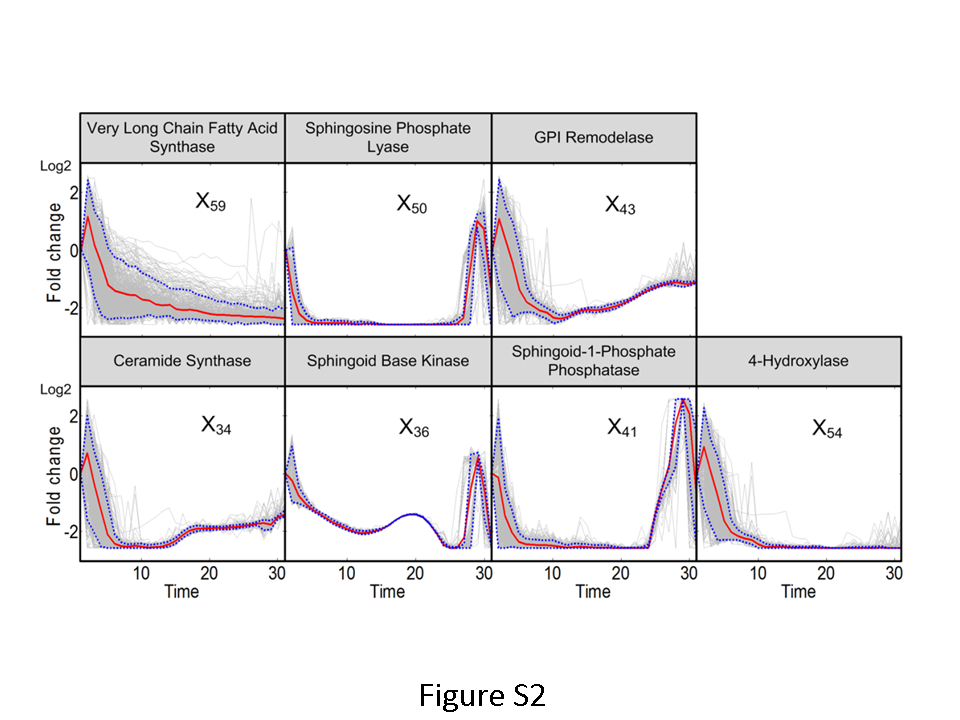

Supplement: Figure S2 — Trends in activities of enzymes in the core region of sphingolipid metabolism. After an initial spike, all enzyme activities in this region are reduced to almost nil. Grey lines are results of 2,000 individual iterations in the large-scale simulation. Red lines are ensemble averages, and dotted blue lines enclose 95% of the results. The figure corresponds to Figure 4 of the main text. (TIF) [file pcbi.1003078.s002.tif]

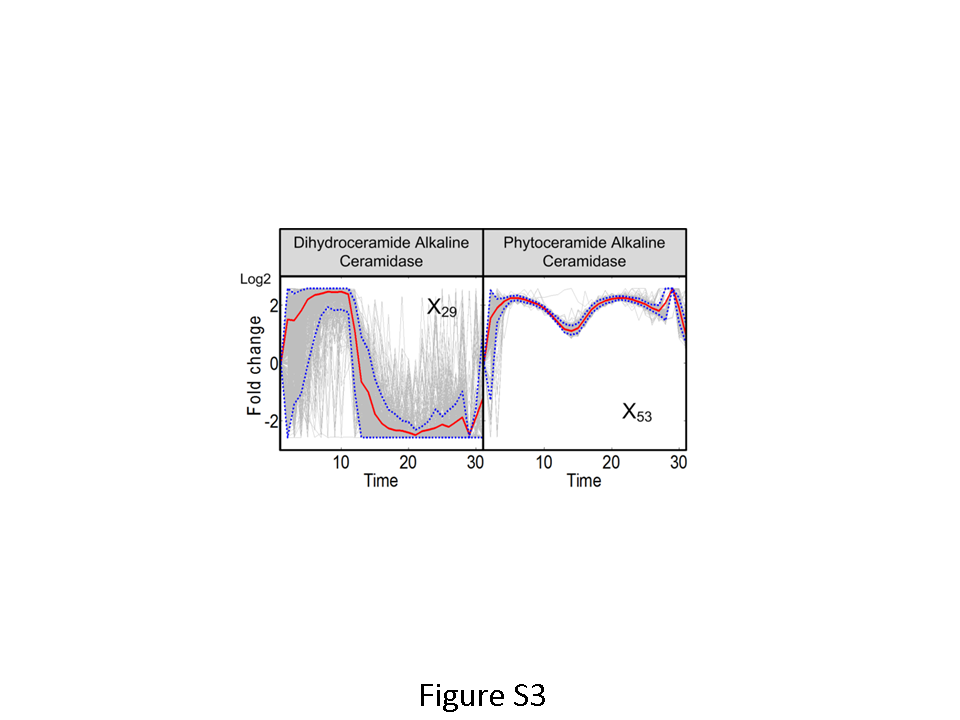

Supplement: Figure S3 — Trends in activities of the two alkaline ceramidases. Dihydroceramide alkaline ceramidase and phytoceramide alkaline ceramidase, which convert the ceramide form into sphingosines, exhibit distinct activity patterns. Grey lines are results of 2,000 individual iterations in the large-scale simulation. Red lines are ensemble averages, and dotted blue lines enclose 95% of the results. The figure corresponds to Figure 5 of the main text. (TIF) [file pcbi.1003078.s003.tif]

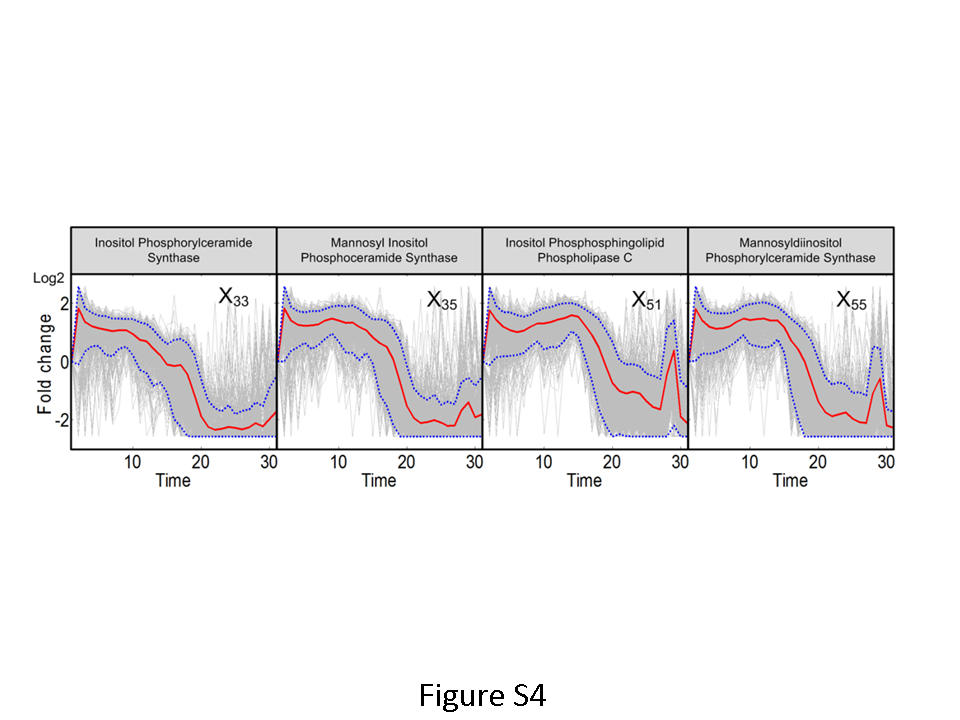

Supplement: Figure S4 — Trends in activities of enzymes associated with complex sphingolipids. Enzymes interconverting complex sphingolipids are at first hyper-active, but tend to lose most activity between 20 and 30 minutes. Grey lines are results of 2,000 individual iterations in the large-scale simulation. Red lines are ensemble averages, and dotted blue lines enclose 95% of the results. The figure corresponds to Figure 6 of the main text. (TIF) [file pcbi.1003078.s004.tif]

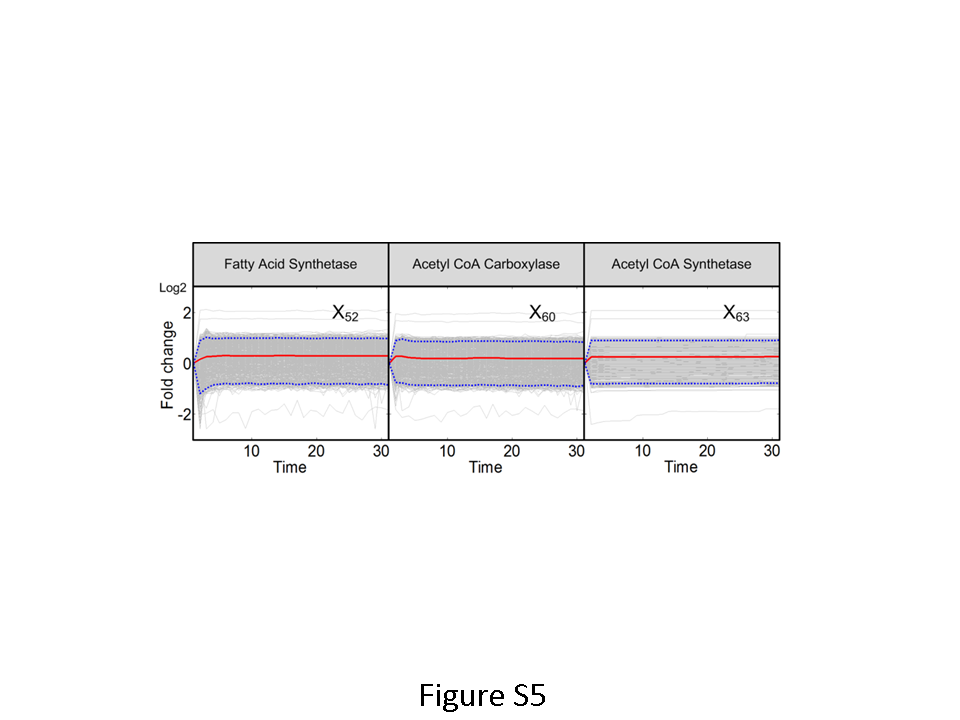

Supplement: Figure S5 — Trends in activities of enzymes associated with fatty acid CoA. The enzymes shown here are responsible for CoA elongation. Grey lines are results of 2,000 individual iterations in the large-scale simulation. Red lines are ensemble averages, and dotted blue lines enclose 95% of the results. The figure corresponds to Figure 7 of the main text. (TIF) [file pcbi.1003078.s005.tif]

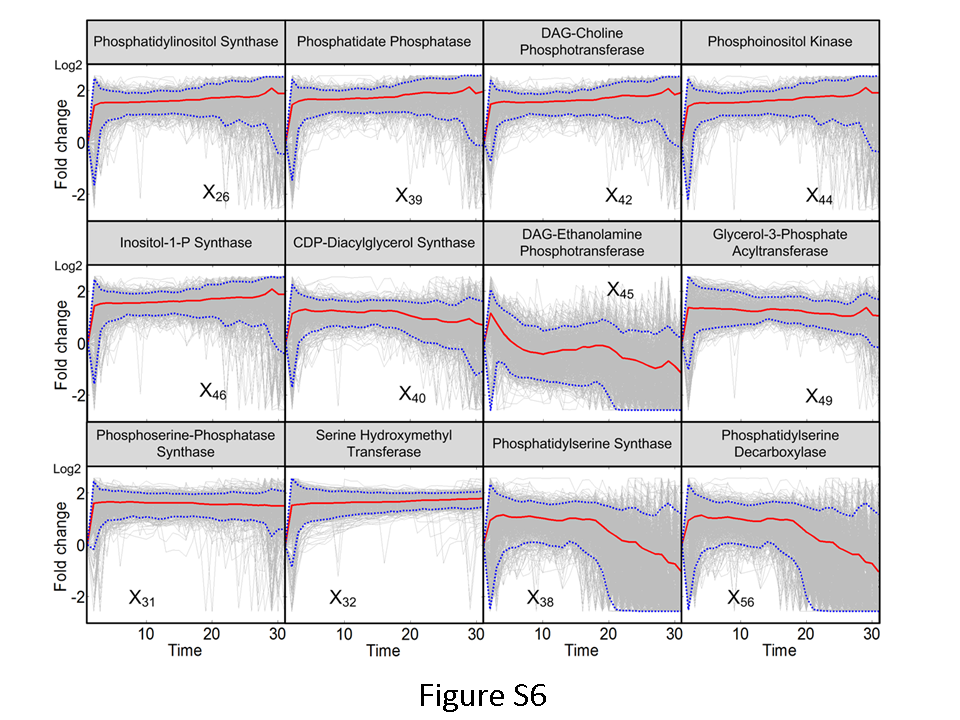

Supplement: Figure S6 — Trends in the remaining enzyme activities. Activities of enzymes at the periphery of the pathway system are not identifiable, mainly due to insufficient information and the fact that these enzymes are also involved in other pathways. Enzymes in two upper panels are related to the phospholipid metabolism and enzymes in the lower panel are related to serine metabolism. Grey lines are results of 2,000 individual iterations in the large-scale simulation. Red lines are averages, and dotted blue lines enclose 95% of the results. The figure corresponds to Figure 8 of the main text. (TIF) [file pcbi.1003078.s006.tif]

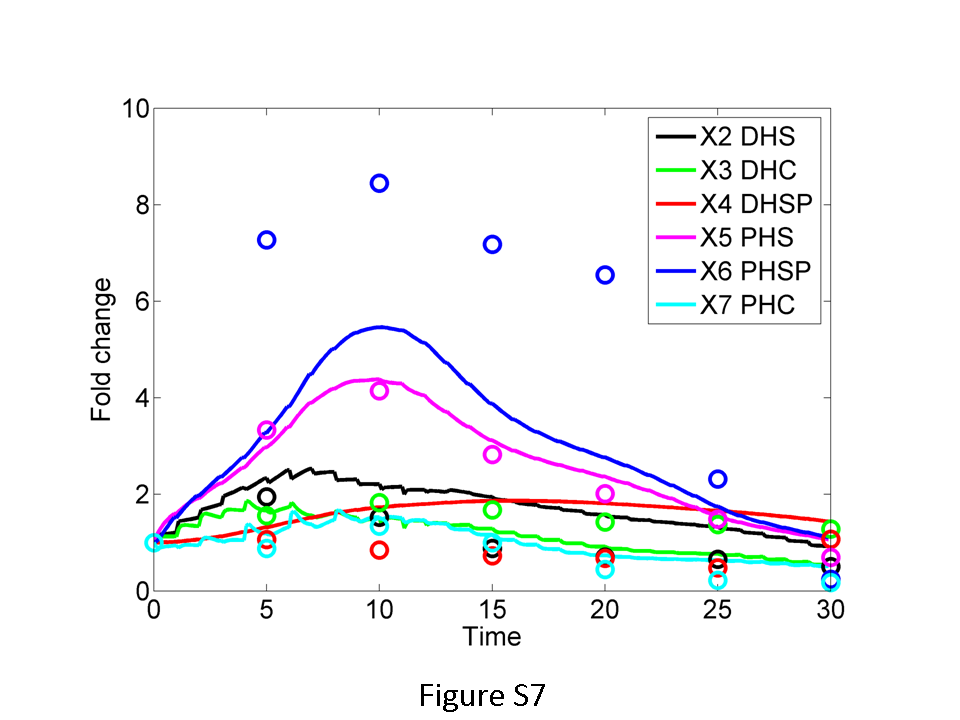

Supplement: Figure S7 — A negative control experiment. When the key enzymes are locked into their normal activity values and all other enzyme activities are allowed to be optimized, the fit of the best model to the experimental data is not very good. (TIF) [file pcbi.1003078.s007.tif]

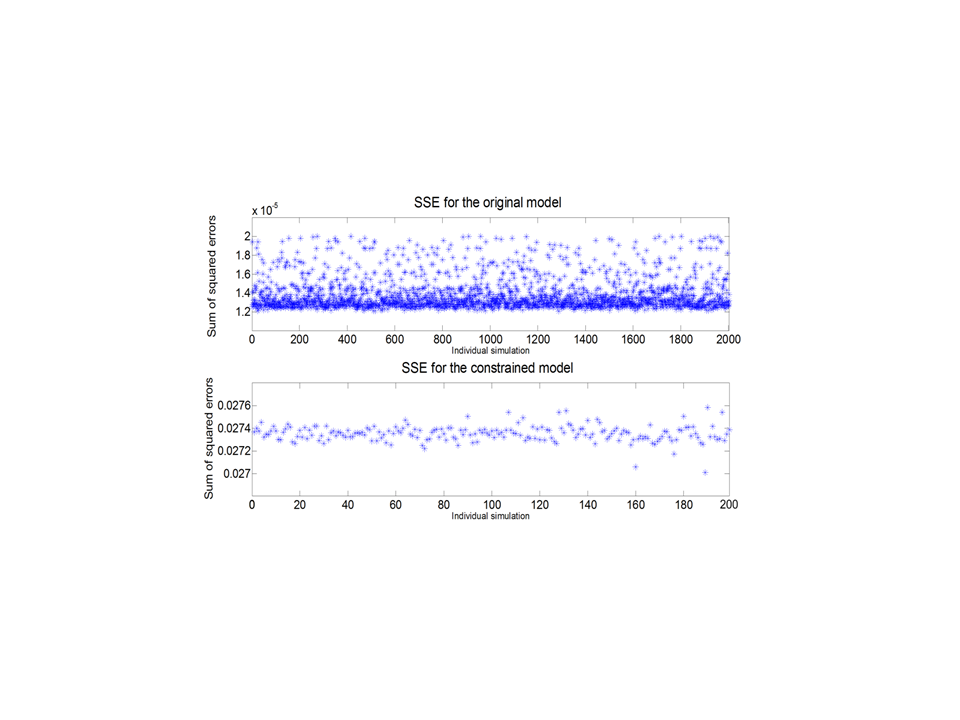

Supplement: Figure S8 — Sums of squared errors for individual optimizations. Upper panel: SSEs for 2,000 simulations with the original model. Lower panel: SSEs for 200 simulations with the constrained model. The X-axis shows the index of each individual simulation, while the Y-axis shows the corresponding sum of squared errors (SSE); note different scales. (TIF) [file pcbi.1003078.s008.tif]

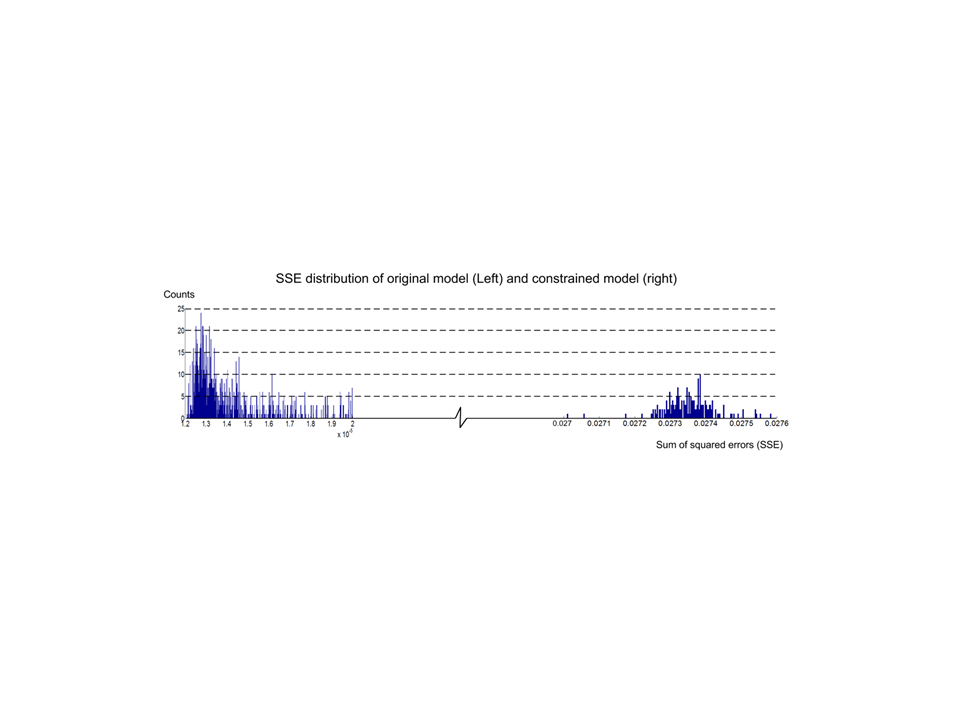

Supplement: Figure S9 — Distributions of sums of squared errors for individual simulations. The distribution on the left contains SSEs for the model in which all enzymes are allowed to change. The distribution on the right contains the corresponding SSE values for the constrained model. (TIF) [file pcbi.1003078.s009.tif]

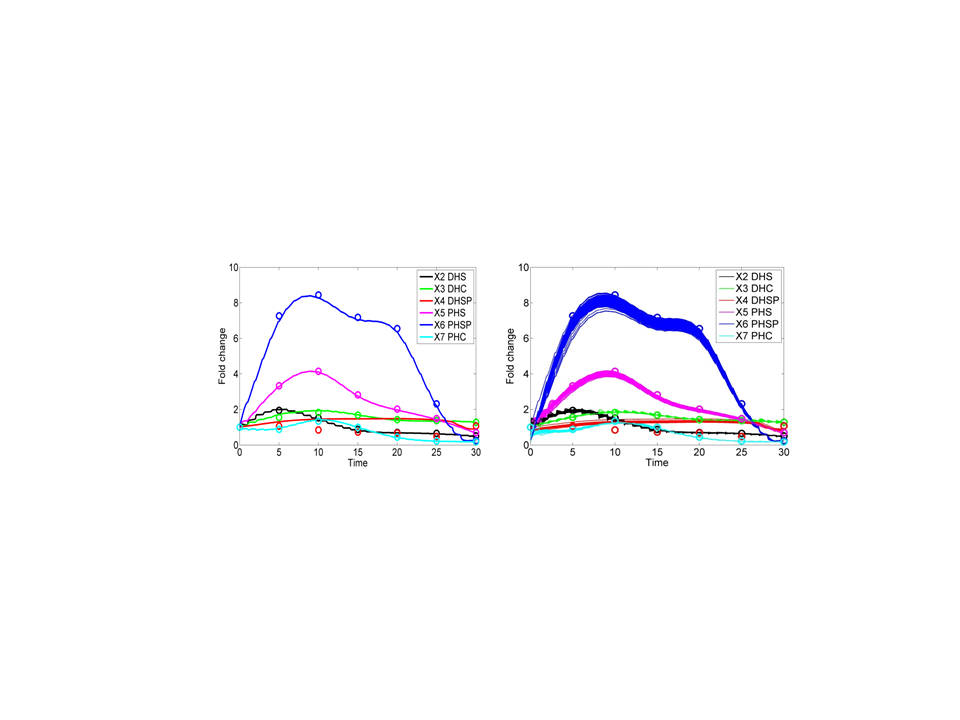

Supplement: Figure S10 — Comparison of data fits. Left panel: Data fitted with the unconstrained averaged model (identical to Figure 1 of the text). Right panel: 179 data fits with individual model simulations that resulted in SSE < 1.25×10^−5^ (cf. Figure S8). (TIF) [file pcbi.1003078.s010.tif]

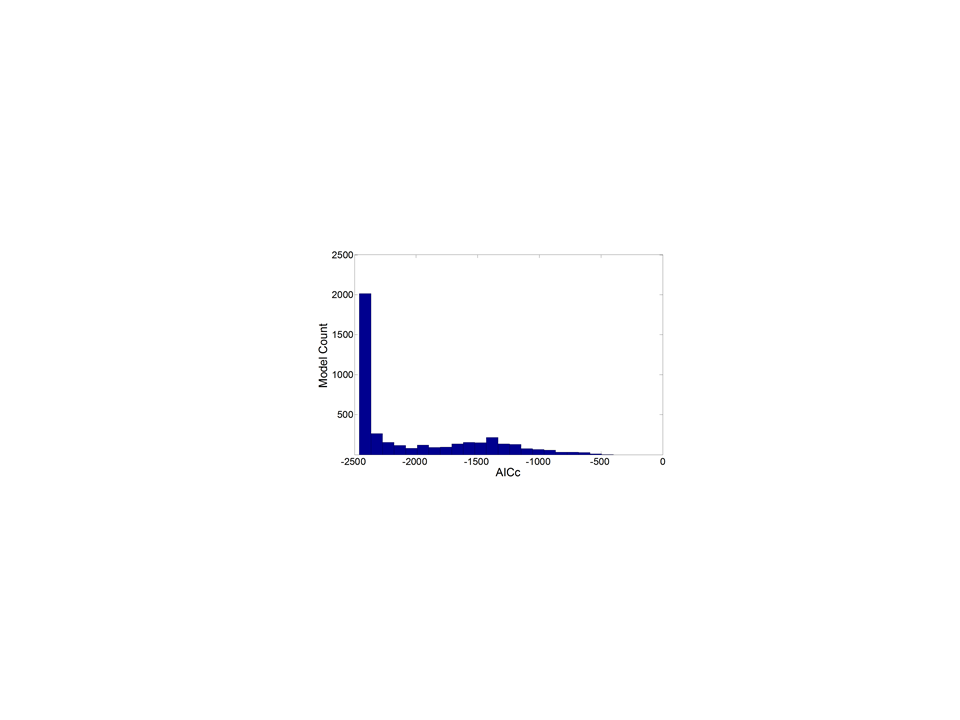

Supplement: Figure S11 — The histogram of AICc values of the 4144 initial models clearly indicates that the 2018 models in the left-most column are superior to all other parameterizations. 99.35% (1991) of the 2004 models identified by SSE fall into this column, thereby demonstrating very strong consistency between the two measures of quality. (TIF) [file pcbi.1003078.s011.tif]
