# Supplementary material for: Coordination of Rapid Sphingolipid Responses to Heat Stress in Yeast
Source: PLoS Comput Biol. 2013 May 30;9(5):e1003078. doi: 10.1371/journal.pcbi.1003078 (PMC3667767; doi:10.1371/journal.pcbi.1003078)
Supplement: Table S1 — Metabolites, enzymes, abbreviations, and variable names. (DOCX) [file pcbi.1003078.s012.docx]

**Table S1: Metabolites, Enzymes, Abbreviations, and Variable Names**

| **Metabolites and their Representations in the Computational Analysis** | | | |
| --- | --- | --- | --- |
| *X*_1_ | 3-Keto-Dihydrosphingosine (KDHS) | *X*_17_ | Cytidine Diphosphate-Ethanolamine (CDP-Eth) |
| *X*_2_ | Dihydrosphingosine (DHS) | *X*_18,_ *X*_18b_ | Mannosylinositol Phosphorylceramide (MIPC-g) from DHC or PHC, respectively |
| *X*_3_ | Dihydroceramide (Dihydro-C) | *X*_19,_ *X*_19b_ | Mannosyldiinositol Phosphorylceramide (M(IP)_2_C-g) from DHC or PHC, respectively |
| *X*_4_ | Dihydrosphingosine-1-P (DHS-P) | *X*_20,_ *X*_20b_ | Plasma Membrane Inositol Phosphorylceramide (IPC-m) from DHC or PHC, respectively |
| *X*_5_ | Phytosphingosine (PHS) | *X*_21,_ *X*_21b_ | Plasma Membrane Mannosylinositol Phosphorylceramide (MIPC-m) from DHC or PHC, respectively |
| *X*_6_ | Phytosphingosine-1-P (PHS-P) | *X*_22,_ *X*_22b_ | Plasma Membrane Mannosyldiinositol Phosphorylceramide (M(IP)_2_C-m) from DHC or PHC, respectively |
| *X*_7_ | Phytoceramide (Phyto-C) | *X*_23_ | Very Long Chain Fatty Acid (C_26_-CoA) |
| *X_8_, X*_8b_ | Inositol Phosphorylceramide (IPC-g) from DHC or PHC, respectively | *X*_24_ | Malonyl-CoA (Mal-CoA) |
| *X*_9_ | CDP-Diacylglycerol (CDP-DAG) | *X*_25_ | Acetyl-CoA (Ac-CoA) |
| *X*_10_ | Phosphatidylserine (PS) | *X*_28_ | Adenosime-5’-Triphosphate (ATP) |
| *X*_11_ | Phosphatidic Acid (PA) | *X*_37_ | 3-Phosphoserine (3-P-Serine) |
| *X*_12_ | Palmitoyl-CoA (Pal-CoA) | *X*_47_ | Glucose-6-P (G6P) |
| *X*_13_ | Serine | *X*_58_ | Palmitate |
| *X*_14_ | Sn-1,2-Diacylglycerol (DAG) | *X*_61_ | CoA |
| *X*_15_ | Phosphatidylinositol (PI) | *X*_62_ | Acetate |
| *X*_16_ | Inositol (I) |  |  |

| **Enzymes and their Representations in the Computational Analysis** | | | |
| --- | --- | --- | --- |
| *X*_26_ | Phosphatidylinositol Synthase (PI Synthase) | *X*_45_ | DG-Ethanolamine Phosphotransferase (EthPT) |
| *X*_27_ | 3-Ketodihydrosphingosine Reductase  (KDHS Reductase) | *X*_46_ | Inositol-1-P Synthase (I-1-P-Synth.) |
| *X*_29_ | Dihydroceramide Alkaline Ceramidase  (Dihydro-CDase) | *X*_48_ | Acyl-CoA-Binding Protein (ACBP) |
| *X*_30_ | Palmitoyl Transport & Palmitoyl-CoA Synthase (Transp./Palmitoyl CoA Synthase) | *X*_49_ | Glycerol-3-Phosphate Acyltransferase (G3P Acyltransferase) |
| *X*_31_ | Phosphoserine-Phosphatase (P-Serine-PPase) | *X*_50_ | Sphingosine-Phosphate Lyase (Lyase) |
| *X*_32_ | Serine Hydroxymethyl Transferase (SHMT) | *X*_51_ | Inositol Phosphosphingolipid Phospholipase C (IPCase) |
| *X*_33_ | Inositol Phosphorylceramide Synthase  (IPC Synthase) | *X*_52_ | Fatty Acid Synthetase (FAS) |
| *X*_34_ | Ceramide Synthase (Cer Synthase) | *X*_53_ | Phytoceramide Alkaline Ceramidase (Phyto-CDase) |
| *X*_35_ | Mannosyl Inositol Phosphoceramide Synthase (MIPC Synthase) | *X*_54_ | 4-Hydroxylase (Hydroxylase; SYR2p-SUR2p) |
| *X*_36_ | Sphingoid Base Kinase | *X*_55_ | Mannosyldiinositol Phosphorylceramide Synthase (M(IP)_2_C Synthase) |
| *X*_38_ | Phosphatidylserine Synthase (PS Synthase) | *X*_56_ | Phosphatidylserine Decarboxylase (PS Decarboxylase) |
| *X*_39_ | Phosphatidate Phosphatase (PA-PPase) | *X*_57_ | Serine Palmitoyltransferase (SPT) |
| *X*_40_ | CDP-Diacylglycerol Synthase (CDP-DAG Synthase) | *X*_59_ | Very Long Chain Fatty Acid Synthase / Elongase (ELO1p) |
| *X*_41_ | Sphingoid-1-phosphate Phosphatase (SB-PPase) | *X*_60_ | Acetyl-Coenzyme A Carboxylase (ACCp) |
| *X*_42_ | DG-Choline Phosphotransferase (ChoPT) | *X*_63_ | Acetyl-Coenzyme A Synthetase (ACSp) |
| *X*_43_ | GPI Remodelase (Remodeling) | *X*_65_ | Not yet identified |
| *X*_44_ | Phosphoinositol Kinase (PI Kinase) | *X*_66_ | Not yet identified |
