# Supplementary material for: Coordination of Rapid Sphingolipid Responses to Heat Stress in Yeast
Source: PLoS Comput Biol. 2013 May 30;9(5):e1003078. doi: 10.1371/journal.pcbi.1003078 (PMC3667767; doi:10.1371/journal.pcbi.1003078)
Supplement: Table S2 — Summary of identifiable dynamic changes in enzyme activities in response to heat stress. (DOCX) [file pcbi.1003078.s013.docx]

**Table S2: Summary of Identifiable Dynamic Changes in Enzyme Activities in Response to Heat Stress**

All enzyme activities initially climb to different degrees, presumably due to the Arrhenius effect. Subsequently, the trends are strikingly different. Numbers represent approximate fold changes in activities, while arrows and colors indicate the direction of change.

| Enzyme | Time  Var. | 0-5 | | | | 5-10 | 10-15 | | 15-20 | | 20-25 | 25-30 | | | | |
| --- | --- | --- | --- | --- | --- | --- | --- | --- | --- | --- | --- | --- | --- | --- | --- | --- |
| 3-keto-dihydrosphingosine reductase | *X*_27_ | ↑ 2.8 | | | | ↓ 1.1 | ↑ 1.2 | | ↓ 1 | | ↓ 0.5 | ↓ 0.4 | | | | |
| Dihydroceramide aklaline ceramidase | *X*_29_ | ↑ 5.5 | | | | | ↔ 0.3 | | | | | ↑ 0.5 | | | | |
| Inositol phosphorylceramide synthase | *X*_33_ | ↑ 3.5 | | | | ↓ 1 | | | ↓0.3 | | | ↑ 0.4 | | | | |
| Ceramide synthase | *X*_34_ | 0-2.5 | | | 2.5-5 | ↔ 0.3 | | | ↑ 0.4 | | | | | | | |
|  |  | ↑ 1.8 | | | ↓ 0.3 |  |  |  |  |  |  |  |  |  |  |  |
| Mannosyl inositol phosphoceramide synthase | *X*_35_ | 0-2.5 | | | 2.5-5 | ↑ 2.8 | ↓ 0.3 | | | | ↔ 0.3 | | | | | |
|  |  | ↑ 3.5 | | | ↓ 2.5 |  |  |  |  |  |  |  |  |  |  |  |
| Sphingoid base kinase | *X*_36_ | 0-2.5 | | | 2.5-5 | ↔ 0.3 | | | | | | 25-28 | | | 28-30 | |
|  |  | ↑ 1.1 | | | ↓ 0.4 |  |  |  |  |  |  | ↑ 1.8 | | | ↓ 0.3 | |
| Sphingoid 1 phosphate phosphatase | *X*_41_ | 0-2.5 | | | 2.5-5 | ↔ 0.3 | | | | | | 25-29 | | | 29-30 | |
|  |  | ↑ 1.2 | | | ↓ 0.3 |  |  |  |  |  |  | ↑ 6 | | | ↓ 0.5 | |
| GPI remodelase | *X*_43_ | 0-2.5 | | | 2.5-5 | ↓ 0.3 | ↔ 0.3 | | | | ↑ 0.4 | ↑ 0.5 | | | | |
|  |  | ↑ 2.2 | | | ↓ 0.4 |  |  |  |  |  |  |  |  |  |  |  |
| Sphingosine phosphate lyase | *X*_50_ | ↓ 0.2 | | | | ↔ 0.3 | | | | | | 25-28 | | | | 28-30 |
|  |  |  |  |  |  |  |  |  |  |  |  | ↑ 2 | | | | ↓ 0.5 |
| Inositol phosphosphingolipid phospholipase C | *X*_51_ | 0-2 | | 2-5 | | ↑ 2.5 | ↑ 3 | | ↓ 0.5 | | ↓ 0.3 | 25-28 | | | 28-30 | |
|  |  | ↑ 3.4 | | ↓ 2.1 | |  |  |  |  |  |  | ↑ 1 | | | ↓ 0.3 | |
| Phytoceramide alkaline ceramidase | *X*_53_ | ↑ 4.5 | | | | ↓ 3.7 | ↓ 2.2 | | ↑ 4.5 | ↓ 3.7 | | 25-28 | | 28-30 | | |
|  |  |  |  |  |  |  |  |  |  |  |  | ↑ 6 | | ↓ 2 | | |
| 4 hydroxylase | *X*_54_ | 0-2 | | | 2-5 | ↔ 0.3 | | | | | | | | | | |
|  |  | ↑ 2 | | | ↓ 0.3 |  |  |  |  |  |  |  |  |  |  |  |
| Mannosyldiinositol phosphorylceramide synthase | *X*_55_ | 0-2 | | | 2-5 | ↑ 2.7 | 10-13 | 13-15 | ↓ 0.5 | | ↓ 0.3 | 25-28 | 28-30 | | | |
|  |  | ↑ 3.5 | | | ↓ 2.2 |  | ↓ 2.6 | ↓ 1.7 |  |  |  | ↑ 0.6 | ↓ 0.3 | | | |
| Serine palmitoyltransferase | *X*_57_ | 0-2 | 2-5 | | | ↓ 0.3 | ↔ 0.3 | | | | | | | | | |
|  |  | ↑ 2.2 | ↓ 0.5 | | |  |  |  |  |  |  |  |  |  |  |  |
| Very long chain fatty acid synthase | *X*_59_ | 0-2 | 2-5 | | | ↓ 0.3 | ↔ 0.3 | | | | | | | | | |
|  |  | ↑ 2.3 | ↓ 0.5 | | |  |  |  |  |  |  |  |  |  |  |  |
