# Supplementary material for: Coordination of Rapid Sphingolipid Responses to Heat Stress in Yeast
Source: PLoS Comput Biol. 2013 May 30;9(5):e1003078. doi: 10.1371/journal.pcbi.1003078 (PMC3667767; doi:10.1371/journal.pcbi.1003078)
Supplement: Table S3 — Estimated Q10 values, based on the initial increases in enzyme activities. (DOCX) [file pcbi.1003078.s014.docx]

**Table S3: Estimated Q_10_ Values, Based on the Initial Increases in Enzyme Activities**

| **Enzyme** | **Variable** | **Q_10_** |
| --- | --- | --- |
| 3-keto-dihydrosphingosine reductase | *X*_27_ | 3.1394 |
| Dihydroceramide aklaline ceramidase | *X*_29_ | *3.0150* |
| Inositol phosphorylceramide synthase | *X*_33_ | *4.0227* |
| Ceramide synthase | *X*_34_ | *1.9215* |
| Mannosyl inositol phosphoceramide synthase | *X*_35_ | *4.0227* |
| Sphingoid base kinase | *X*_36_ | *1.1117* |
| Sphingoid 1 phosphate phosphatase | *X*_41_ | *1.2246* |
| GPI remodelase | *X*_43_ | *2.4014* |
| Sphingosine phosphate lyase | *X*_50_ | *0.1673* |
| Inositol phosphosphingolipid phospholipase C | *X*_51_ | *3.8952* |
| Phytoceramide alkaline ceramidase | *X*_53_ | *3.5153* |
| Hydroxylase | *X*_54_ | *2.1601* |
| Mannosyldiinositol phosphorylceramide synthase | *X*_55_ | *4.0227* |
| Serine palmitoyltransferase | *X*_57_ | *2.4014* |
| Very long chain fatty acid synthase | *X*_59_ | *2.5230* |
