# Supplementary material for: Coordination of Rapid Sphingolipid Responses to Heat Stress in Yeast
Source: PLoS Comput Biol. 2013 May 30;9(5):e1003078. doi: 10.1371/journal.pcbi.1003078 (PMC3667767; doi:10.1371/journal.pcbi.1003078)
Supplement: Text S1 — This file contains model equations, model details in Tables S1, S2, S3, details regarding the optimization procedure, an alternative representation (Log2) of trends in enzyme activities, an estimation of Q10 values for enzymes of the sphingolipid pathway, an assessment of simulation results with a “negative control,” comments on the Akaike Information Criterion, and additional references. (DOCX) [file pcbi.1003078.s016.docx]

Coordination of Rapid Sphingolipid Responses to

Heat Stress in Yeast

Po-Wei Chen, Luis L. Fonseca, Yusuf A. Hannun and Eberhard O. Voit

**Supplementary Information: Text S1**

***Model Equations***

The model equations (Eq. S1) were adopted from Alvarez *et al.* [[1](#_ENREF_1),[2](#_ENREF_2)]. One exception was the use of a single representation of IPCase rather than two in the original papers. Furthermore, we expanded the model from the original IPC mechanism, consisting of reversible reactions between the IPC family and ceramides, to a refined mechanism that distinguished between dihydroceramide and phytoceramide forms. To achieve this new mechanism, we included six more variables (*X*_8b_, *X*_18b_ – *X*_22b_) into the system to represent the IPC family as it reacts with phytoceramide, while leaving the original IPC variables (*X*_8_, *X*_18_ – *X*_22_) to represent the IPC family reacting with dihydroceramide. The corresponding equations are given below; please refer to Table S1 for definitions of variables.

$$\frac{dX_{1}}{dt}=\gamma_{11}X_{12}^{f_{1,12,1}}X_{13}^{f_{1,13,2}}X_{57}^{f_{1,57,3}}-\gamma_{12}X_{1}^{f_{1,1,4}}X_{27}^{f_{1,27,5}}$$

$$\frac{dX_{2}}{dt}=\gamma_{21}X_{1}^{f_{2,1,1}}X_{27}^{f_{2,27,2}}+\gamma_{22}X_{3}^{f_{2,3,3}}X_{29}^{f_{2,29,4}}+\gamma_{23}X_{4}^{f_{2,4,5}}X_{41}^{f_{2,41,6}}-\gamma_{24}X_{2}^{f_{2,2,7}}X_{23}^{f_{2,23,8}}X_{34}^{f_{2,34,9}}-\gamma_{25}X_{2}^{f_{2,2,10}}X_{28}^{f_{2,28,11}}X_{36}^{f_{2,36,12}}-\gamma_{26}X_{2}^{f_{2,2,13}}X_{54}^{f_{2,54,14}}$$

$$\frac{dX_{3}}{dt}=\gamma_{31}X_{2}^{f_{3,2,1}}X_{23}^{f_{3,23,2}}X_{34}^{f_{3,34,3}}+\gamma_{32}X_{8}^{f_{3,8,4}}X_{51}^{f_{3,51,5}}+\gamma_{33}X_{18}^{f_{3,18,6}}X_{51}^{f_{3,51,7}}+\gamma_{34}X_{19}^{f_{3,19,8}}X_{51}^{f_{3,51,9}}-\gamma_{35}X_{3}^{f_{3,3,10}}X_{29}^{f_{3,29,11}}-\gamma_{36}X_{3}^{f_{3,3,12}}X_{54}^{f_{3,54,13}}-\gamma_{37}X_{2}^{f_{3,2,14}}X_{3}^{f_{3,3,15}}X_{5}^{f_{3,5,16}}X_{15}^{f_{3,15,17}}X_{33}^{f_{3,33,18}}$$

$$\frac{dX_{4}}{dt}=\gamma_{41}X_{2}^{f_{4,2,1}}X_{28}^{f_{4,28,2}}X_{36}^{f_{4,36,3}}-\gamma_{42}X_{4}^{f_{4,4,4}}X_{41}^{f_{4,41,5}}-\gamma_{43}X_{4}^{f_{4,4,6}}X_{50}^{f_{4,50,7}}$$

$$\frac{dX_{5}}{dt}=\gamma_{51}X_{2}^{f_{5,2,1}}X_{54}^{f_{5,54,2}}+\gamma_{52}X_{6}^{f_{5,6,3}}X_{41}^{f_{5,41,4}}+\gamma_{53}X_{7}^{f_{5,7,5}}X_{53}^{f_{5,53,6}}-\gamma_{54}X_{5}^{f_{5,5,7}}X_{23}^{f_{5,23,8}}X_{34}^{f_{5,34,9}}-\gamma_{55}X_{5}^{f_{5,5,10}}X_{28}^{f_{5,28,11}}X_{36}^{f_{5,36,12}}$$

$$\frac{dX_{6}}{dt}=\gamma_{41}X_{2}^{f_{4,2,1}}X_{28}^{f_{4,28,2}}X_{36}^{f_{4,36,3}}-\gamma_{42}X_{4}^{f_{4,4,4}}X_{41}^{f_{4,41,5}}-\gamma_{43}X_{4}^{f_{4,4,6}}X_{50}^{f_{4,50,7}}$$

$$\frac{dX_{7}}{dt}=\gamma_{71}X_{2}^{f_{7,2,1}}X_{23}^{f_{7,23,2}}X_{34}^{f_{7,34,3}}+\gamma_{72}X_{8b}^{f_{7,8b,4}}X_{51}^{f_{7,51,5}}+\gamma_{73}X_{3}^{f_{7,3,6}}X_{54}^{f_{7,54,7}}+\gamma_{74}X_{18b}^{f_{7,18b,8}}X_{51}^{f_{7,51,9}}+\gamma_{75}X_{19b}^{f_{7,19b,10}}X_{51}^{f_{7,51,11}}-\gamma_{76}X_{7}^{f_{7,7,12}}X_{53}^{f_{7,53,13}}-\gamma_{77}X_{2}^{f_{7,2,14}}X_{5}^{f_{7,5,15}}X_{7}^{f_{7,7,16}}X_{15}^{f_{7,15,17}}X_{33}^{f_{7,33,18}}-\gamma_{78}X_{7}^{f_{7,7,19}}X_{43}^{f_{7,43,20}}$$

$$\frac{dX_{8}}{dt}=\gamma_{82}X_{2}^{f_{8,2,6}}X_{3}^{f_{8,3,7}}X_{5}^{f_{8,5,8}}X_{15}^{f_{8,15,9}}X_{33}^{f_{8,33,10}}+\gamma_{83}X_{20}^{f_{8,20,11}}-\gamma_{85}X_{8}^{f_{8,8,14}}X_{35}^{f_{8,35,15}}-\gamma_{86}X_{8}^{f_{8,8,16}}X_{51}^{f_{8,51,17}}-\gamma_{87}X_{8}^{f_{8,8,18}}$$

$$\frac{dX_{9}}{dt}=\gamma_{91}X_{11}^{f_{9,11,1}}X_{40}^{f_{9,40,2}}-\gamma_{92}X_{2}^{f_{9,2,3}}X_{5}^{f_{9,5,4}}X_{9}^{f_{9,9,5}}X_{11}^{f_{9,11,6}}X_{13}^{f_{9,13,7}}X_{14}^{f_{9,14,8}}X_{15}^{f_{9,15,9}}X_{16}^{f_{9,16,10}}X_{38}^{f_{9,38,11}}-\gamma_{93}X_{9}^{f_{9,9,12}}X_{16}^{f_{9,16,13}}X_{26}^{f_{9,26,14}}$$

$$\frac{dX_{10}}{dt}=\gamma_{10,1}X_{2}^{f_{10,2,1}}X_{5}^{f_{10,5,2}}X_{9}^{f_{10,9,3}}X_{11}^{f_{10,11,4}}X_{13}^{f_{10,13,5}}X_{14}^{f_{10,14,6}}X_{15}^{f_{10,15,7}}X_{16}^{f_{10,16,8}}X_{38}^{f_{10,38,9}}-\gamma_{10,2}X_{10}^{f_{10,10,10}}X_{56}^{f_{10,56,11}}$$

$$\frac{dX_{11}}{dt}=\gamma_{11,1}X_{10}^{f_{11,10,1}}X_{12}^{f_{11,12,2}}X_{49}^{f_{11,49,3}}-\gamma_{11,2}X_{2}^{f_{11,2,4}}X_{5}^{f_{11,5,5}}X_{9}^{f_{11,9,6}}X_{11}^{f_{11,11,7}}X_{15}^{f_{11,15,8}}X_{39}^{f_{11,39,9}}-\gamma_{11,3}X_{11}^{f_{11,11,10}}X_{40}^{f_{11,40,11}}$$

$$\frac{dX_{12}}{dt}=\gamma_{12,1}X_{30}^{f_{12,30,1}}X_{58}^{f_{12,58,2}}+\gamma_{12,2}X_{24}^{f_{12,24,3}}X_{25}^{f_{12,25,4}}X_{52}^{f_{12,52,5}}+\gamma_{12,3}X_{4}^{f_{12,4,6}}X_{50}^{f_{12,50,7}}+\gamma_{12,4}X_{6}^{f_{12,6,8}}X_{50}^{f_{12,50,9}}-\gamma_{12,5}X_{12}^{f_{12,12,10}}X_{13}^{f_{12,13,11}}X_{57}^{f_{12,57,12}}-\gamma_{12,6}X_{10}^{f_{12,10,13}}X_{12}^{f_{12,12,14}}X_{49}^{f_{12,49,15}}-\gamma_{12,7}X_{12}^{f_{12,12,16}}X_{48}^{f_{12,48,17}}-\gamma_{12,8}X_{12}^{f_{12,12,18}}X_{24}^{f_{12,24,19}}X_{59}^{f_{12,59,20}}$$

$$\frac{dX_{13}}{dt}=\gamma_{13,1}X_{31}^{f_{13,31,1}}X_{37}^{f_{13,37,2}}+\gamma_{13,2}X_{65}^{f_{13,65,3}}X_{66}^{f_{13,66,4}}-\gamma_{13,3}X_{2}^{f_{13,2,5}}X_{5}^{f_{13,5,6}}X_{9}^{f_{13,9,7}}X_{11}^{f_{13,5,8}}X_{13}^{f_{13,13,9}}X_{14}^{f_{13,14,10}}X_{15}^{f_{13,15,11}}X_{16}^{f_{13,16,12}}X_{38}^{f_{13,38,13}}-\gamma_{13,4}X_{12}^{f_{13,12,14}}X_{13}^{f_{13,13,15}}X_{57}^{f_{13,57,16}}-\gamma_{13,5}X_{13}^{f_{13,13,17}}X_{32}^{f_{13,32,18}}$$

$$\frac{dX_{14}}{dt}=\gamma_{14,1}X_{2}^{f_{14,2,1}}X_{5}^{f_{14,5,2}}X_{9}^{f_{14,9,3}}X_{11}^{f_{14,11,4}}X_{15}^{f_{14,15,5}}X_{39}^{f_{14,39,6}}+\gamma_{14,2}X_{2}^{f_{14,2,7}}X_{5}^{f_{14,5,8}}X_{7}^{f_{14,7,9}}X_{15}^{f_{14,15,10}}X_{33}^{f_{14,33,11}}+\gamma_{14,3}X_{2}^{f_{14,2,12}}X_{3}^{f_{14,3,13}}X_{5}^{f_{14,5,14}}X_{15}^{f_{14,15,15}}X_{33}^{f_{14,33,16}}+\gamma_{14,4}X_{15}^{f_{14,15,17}}X_{18}^{f_{14,18,18}}X_{55}^{f_{14,55,19}}-\gamma_{14,5}X_{14}^{f_{14,14,20}}X_{17}^{f_{14,17,21}}X_{45}^{f_{14,45,22}}-\gamma_{14,6}X_{14}^{f_{14,14,23}}X_{42}^{f_{14,42,24}}$$

$$\frac{dX_{15}}{dt}=\gamma_{15,1}X_{9}^{f_{15,9,1}}X_{16}^{f_{15,16,2}}X_{26}^{f_{15,26,3}}-\gamma_{15,2}X_{2}^{f_{15,2,4}}X_{5}^{f_{15,5,5}}X_{7}^{f_{15,7,6}}X_{15}^{f_{15,15,7}}X_{33}^{f_{15,33,8}}-\gamma_{15,3}X_{2}^{f_{15,2,9}}X_{3}^{f_{15,3,10}}X_{5}^{f_{15,5,11}}X_{15}^{f_{15,15,12}}X_{33}^{f_{15,33,13}}-\gamma_{15,4}X_{15}^{f_{15,15,14}}X_{28}^{f_{15,28,15}}X_{44}^{f_{15,44,16}}-\gamma_{15,5}X_{15}^{f_{15,15,17}}X_{18}^{f_{15,18,18}}X_{55}^{f_{15,55,19}}$$

(S1)

$$\frac{dX_{16}}{dt}=\gamma_{16,1}X_{46}^{f_{16,46,1}}X_{47}^{f_{16,47,2}}-\gamma_{16,2}X_{9}^{f_{16,9,3}}X_{16}^{f_{16,16,4}}X_{26}^{f_{16,26,5}}$$

$$\frac{dX_{17}}{dt}=\gamma_{17,1}X_{4}^{f_{17,4,1}}X_{50}^{f_{17,50,2}}+\gamma_{17,2}X_{6}^{f_{17,6,3}}X_{50}^{f_{17,50,4}}-\gamma_{17,3}X_{14}^{f_{17,14,5}}X_{17}^{f_{17,17,6}}X_{45}^{f_{17,45,7}}$$

$$\frac{dX_{18}}{dt}=\gamma_{18,1}X_{8}^{f_{18,8,1}}X_{35}^{f_{18,35,2}}+\gamma_{18,2}X_{21}^{f_{18,21,3}}-\gamma_{18,3}X_{18}^{f_{18,18,4}}X_{51}^{f_{18,51,5}}-\gamma_{18,5}X_{15}^{f_{18,15,8}}X_{18}^{f_{18,18,9}}X_{55}^{f_{18,55,10}}-\gamma_{18,6}X_{18}^{f_{18,18,11}}$$

$$\frac{dX_{19}}{dt}=\gamma_{19,1}X_{15}^{f_{19,15,1}}X_{18}^{f_{19,18,2}}X_{55}^{f_{19,55,3}}+\gamma_{19,2}X_{22}^{f_{19,22,4}}-\gamma_{19,3}X_{19}^{f_{19,19,5}}X_{51}^{f_{19,51,6}}-\gamma_{19,5}X_{19}^{f_{19,19,9}}$$

$$\frac{dX_{20}}{dt}=\gamma_{20,1}X_{8}^{f_{20,8,1}}-\gamma_{20,2}X_{20}^{f_{20,20,2}}$$

$$\frac{dX_{21}}{dt}=\gamma_{21,1}X_{18}^{f_{21,18,1}}-\gamma_{21,2}X_{21}^{f_{21,21,2}}$$

$$\frac{dX_{22}}{dt}=\gamma_{22,1}X_{19}^{f_{22,19,1}}-\gamma_{22,2}X_{22}^{f_{22,22,2}}$$

$$\frac{dX_{23}}{dt}=\gamma_{23,1}X_{12}^{f_{23,12,1}}X_{24}^{f_{23,24,2}}X_{59}^{f_{23,59,3}}-\gamma_{23,2}X_{2}^{f_{23,2,4}}X_{23}^{f_{23,23,5}}X_{34}^{f_{23,34,6}}-\gamma_{23,3}X_{5}^{f_{23,5,7}}X_{23}^{f_{23,23,8}}X_{34}^{f_{23,34,9}}$$

$$\frac{dX_{24}}{dt}=\gamma_{24,1}X_{12}^{f_{24,12,1}}X_{23}^{f_{24,23,2}}X_{25}^{f_{24,25,3}}X_{28}^{f_{24,28,4}}X_{60}^{f_{24,60,5}}-\gamma_{24,2}X_{12}^{f_{24,12,6}}X_{24}^{f_{24,24,7}}X_{59}^{f_{24,59,8}}-\gamma_{24,3}X_{24}^{f_{24,24,9}}X_{25}^{f_{24,25,10}}X_{52}^{f_{24,52,11}}$$

$$\frac{dX_{25}}{dt}=\gamma_{25,1}X_{12}^{f_{25,12,1}}X_{28}^{f_{25,28,2}}X_{61}^{f_{25,61,3}}X_{62}^{f_{25,62,4}}X_{63}^{f_{25,63,5}}-\gamma_{25,2}X_{12}^{f_{25,12,6}}X_{23}^{f_{25,23,7}}X_{25}^{f_{25,25,8}}X_{28}^{f_{25,28,9}}X_{60}^{f_{25,60,10}}-\gamma_{25,3}X_{24}^{f_{25,24,11}}X_{25}^{f_{25,25,12}}X_{52}^{f_{25,52,13}}$$

$$\frac{dX_{8b}}{dt}=\gamma_{26,1}X_{2}^{f_{26,2,1}}X_{5}^{f_{26,5,2}}X_{7}^{f_{26,7,3}}X_{15}^{f_{26,15,4}}X_{33}^{f_{26,33,5}}+\gamma_{26,2}X_{20b}^{f_{26,20b,6}}-\gamma_{26,3}X_{8b}^{f_{26,8b,7}}X_{35}^{f_{26,35,8}}-\gamma_{26,4}X_{8b}^{f_{26,8b,9}}X_{51}^{f_{26,51,10}}-\gamma_{26,5}X_{8b}^{f_{26,8b,11}}$$

$$\frac{dX_{18b}}{dt}=\gamma_{27,1}X_{8b}^{f_{27,8b,1}}X_{35}^{f_{27,35,2}}+\gamma_{27,2}X_{21b}^{f_{27,21b,3}}-\gamma_{18,3}X_{18b}^{f_{27,18b,4}}X_{51}^{f_{27,51,5}}-\gamma_{18,4}X_{15}^{f_{27,15,6}}X_{18b}^{f_{27,18b,7}}X_{55}^{f_{27,55,8}}-\gamma_{18,5}X_{18b}^{f_{27,18b,9}}$$

$$\frac{dX_{19b}}{dt}=\gamma_{28,1}X_{15}^{f_{28,15,1}}X_{18b}^{f_{28,18b,2}}X_{55}^{f_{28,55,3}}+\gamma_{28,2}X_{22b}^{f_{28,22b,4}}-\gamma_{28,3}X_{19b}^{f_{28,19b,5}}X_{51}^{f_{28,51,6}}-\gamma_{28,4}X_{19b}^{f_{28,19b,7}}$$

$$\frac{dX_{20b}}{dt}=\gamma_{29,1}X_{8b}^{f_{29,8b,1}}-\gamma_{29,2}X_{20b}^{f_{29,20b,2}}$$

$$\frac{dX_{21b}}{dt}=\gamma_{30,1}X_{18b}^{f_{30,18b,1}}-\gamma_{30,2}X_{21b}^{f_{30,21b,2}}$$

$$\frac{dX_{22b}}{dt}=\gamma_{31,1}X_{19b}^{f_{31,19b,1}}-\gamma_{31,2}X_{22b}^{f_{31,22b,2}}$$

**Table S1: Metabolites, Enzymes, Abbreviations, and Variable Names**

| **Metabolites and their Representations in the Computational Analysis** | | | |
| --- | --- | --- | --- |
| *X*_1_ | 3-Keto-Dihydrosphingosine (KDHS) | *X*_17_ | Cytidine Diphosphate-Ethanolamine (CDP-Eth) |
| *X*_2_ | Dihydrosphingosine (DHS) | *X*_18,_ *X*_18b_ | Mannosylinositol Phosphorylceramide (MIPC-g) from DHC or PHC, respectively |
| *X*_3_ | Dihydroceramide (Dihydro-C) | *X*_19,_ *X*_19b_ | Mannosyldiinositol Phosphorylceramide (M(IP)_2_C-g) from DHC or PHC, respectively |
| *X*_4_ | Dihydrosphingosine-1-P (DHS-P) | *X*_20,_ *X*_20b_ | Plasma Membrane Inositol Phosphorylceramide (IPC-m) from DHC or PHC, respectively |
| *X*_5_ | Phytosphingosine (PHS) | *X*_21,_ *X*_21b_ | Plasma Membrane Mannosylinositol Phosphorylceramide (MIPC-m) from DHC or PHC, respectively |
| *X*_6_ | Phytosphingosine-1-P (PHS-P) | *X*_22,_ *X*_22b_ | Plasma Membrane Mannosyldiinositol Phosphorylceramide (M(IP)_2_C-m) from DHC or PHC, respectively |
| *X*_7_ | Phytoceramide (Phyto-C) | *X*_23_ | Very Long Chain Fatty Acid (C_26_-CoA) |
| *X_8_, X*_8b_ | Inositol Phosphorylceramide (IPC-g) from DHC or PHC, respectively | *X*_24_ | Malonyl-CoA (Mal-CoA) |
| *X*_9_ | CDP-Diacylglycerol (CDP-DAG) | *X*_25_ | Acetyl-CoA (Ac-CoA) |
| *X*_10_ | Phosphatidylserine (PS) | *X*_28_ | Adenosime-5’-Triphosphate (ATP) |
| *X*_11_ | Phosphatidic Acid (PA) | *X*_37_ | 3-Phosphoserine (3-P-Serine) |
| *X*_12_ | Palmitoyl-CoA (Pal-CoA) | *X*_47_ | Glucose-6-P (G6P) |
| *X*_13_ | Serine | *X*_58_ | Palmitate |
| *X*_14_ | Sn-1,2-Diacylglycerol (DAG) | *X*_61_ | CoA |
| *X*_15_ | Phosphatidylinositol (PI) | *X*_62_ | Acetate |
| *X*_16_ | Inositol (I) |  |  |

| **Enzymes and their Representations in the Computational Analysis** | | | |
| --- | --- | --- | --- |
| *X*_26_ | Phosphatidylinositol Synthase (PI Synthase) | *X*_45_ | DG-Ethanolamine Phosphotransferase (EthPT) |
| *X*_27_ | 3-Ketodihydrosphingosine Reductase  (KDHS Reductase) | *X*_46_ | Inositol-1-P Synthase (I-1-P-Synth.) |
| *X*_29_ | Dihydroceramide Alkaline Ceramidase  (Dihydro-CDase) | *X*_48_ | Acyl-CoA-Binding Protein (ACBP) |
| *X*_30_ | Palmitoyl Transport & Palmitoyl-CoA Synthase (Transp./Palmitoyl CoA Synthase) | *X*_49_ | Glycerol-3-Phosphate Acyltransferase (G3P Acyltransferase) |
| *X*_31_ | Phosphoserine-Phosphatase (P-Serine-PPase) | *X*_50_ | Sphingosine-Phosphate Lyase (Lyase) |
| *X*_32_ | Serine Hydroxymethyl Transferase (SHMT) | *X*_51_ | Inositol Phosphosphingolipid Phospholipase C (IPCase) |
| *X*_33_ | Inositol Phosphorylceramide Synthase  (IPC Synthase) | *X*_52_ | Fatty Acid Synthetase (FAS) |
| *X*_34_ | Ceramide Synthase (Cer Synthase) | *X*_53_ | Phytoceramide Alkaline Ceramidase (Phyto-CDase) |
| *X*_35_ | Mannosyl Inositol Phosphoceramide Synthase (MIPC Synthase) | *X*_54_ | 4-Hydroxylase (Hydroxylase; SYR2p-SUR2p) |
| *X*_36_ | Sphingoid Base Kinase | *X*_55_ | Mannosyldiinositol Phosphorylceramide Synthase (M(IP)_2_C Synthase) |
| *X*_38_ | Phosphatidylserine Synthase (PS Synthase) | *X*_56_ | Phosphatidylserine Decarboxylase (PS Decarboxylase) |
| *X*_39_ | Phosphatidate Phosphatase (PA-PPase) | *X*_57_ | Serine Palmitoyltransferase (SPT) |
| *X*_40_ | CDP-Diacylglycerol Synthase (CDP-DAG Synthase) | *X*_59_ | Very Long Chain Fatty Acid Synthase / Elongase (ELO1p) |
| *X*_41_ | Sphingoid-1-phosphate Phosphatase (SB-PPase) | *X*_60_ | Acetyl-Coenzyme A Carboxylase (ACCp) |
| *X*_42_ | DG-Choline Phosphotransferase (ChoPT) | *X*_63_ | Acetyl-Coenzyme A Synthetase (ACSp) |
| *X*_43_ | GPI Remodelase (Remodeling) | *X*_65_ | Not yet identified |
| *X*_44_ | Phosphoinositol Kinase (PI Kinase) | *X*_66_ | Not yet identified |

***Optimization Procedure***

The optimization strategy of our approach can be broken down into several steps:

1. Fix rate constants and kinetic orders in the GMA model, as reported in[[1](#_ENREF_1)].
2. Set upper and lower bounds for changes in enzyme activities.
3. Acquire smoothed heat stress metabolite measurements at time point *t*, where *t* = 1 … 30.
4. At time point *t*, minimize the norm between the smoothed data and the simulation results of the GMA model with appropriate weights, as indicated in Eq. (S2), that give each metabolite equal importance. For the case *t* = 1, assign as initial guess the normal baseline value. For all other time points, assign the initial guess of each independent variable (enzyme) to the corresponding value in the previous solution. Thus, at each time point execute the following minimization:

(S2)

1. Check the GMA simulation results for each iteration. If the GMA model produces negative or imaginary values for any of the dependent variables, then randomize the initial guess of the independent variables and return to step 4. Continue with step 5 until the GMA simulation produces reasonable (positive) values.
2. Collect the solutions of independent variables (enzymes) for the given time point. If *t* = 30, terminate.
3. Execute this iteration many (4144) times.

We randomly sampled the initial states (at the one-minute time point after the beginning of heat stress) of enzymatic activities in four-fold ranges (two-fold up and down) with respect to the normal steady-state values of enzyme activities. For example, the steady state for ceramide synthase is 1.65e-5 μM/min/mg, so that the initial guess for this enzyme was sampled from the range [0.825e-5, 3.3e-5]. This four-fold range only refers to the initial point. It is to be considered rather wide, because only one minute earlier the system had resided at its nominal steady state (under optimal temperature conditions). It would therefore seem unrealistic to allow for, say, 10 or 100-fold changes. Once the system was initialized, the enzyme activities were allowed to vary much further from their optimal activity levels, namely within a 12-fold range.

This setting of 12-fold activity ranges was inspired by experimental data showing that yeast seems to respond to stress by changing many enzyme activities moderately, rather than changing a few key enzymes very strongly. At least this strategy was observed in the sphingolipid response to the diauxic shift in yeast [[3](#_ENREF_3)]. In addition to this heuristic rationale, extensive preliminary testing suggested upper and lower bounds for all enzyme activities of about 6 times and 1/6 times the baseline levels. Modest variations in these bounds (to 10 and 1/10) were not influential, whereas bounds selected too small (2 and 1/2) did not allow enough flexibility and led to inferior minimization results, while bounds selected much larger (100 and 1/100) created solutions that appeared to be unrealistic.

For the minimization we used the Active Set Algorithm implemented in Matlab. This method is a local optimization algorithm that is based on Lagrange multipliers. While it is mathematically possible that a local algorithm would miss other acceptable solutions, it seems in our case biologically reasonable to search for enzyme profiles in moderately wide neighborhoods of their normal activity states, which can be expected to correspond to the basin of attraction of the local algorithm. We preferred a local algorithm over one of the evolutionary algorithms, because the latter, while excellent for global searches, sometimes have problems identifying good solutions within moderate ranges.

***Log_2_ Representation of Trends in Enzyme Activities***

In order to provide greater resolution for reduced enzyme activities, Figures S1 to S6 show all simulation results with a log_2_ scale. These figures correspond to Figures 3 to 8 in the main text.


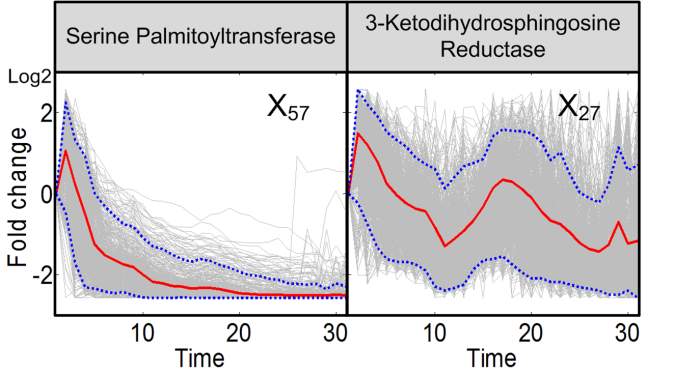


**FIGURE S1. Trends in activities of enzyme at the entry point of sphingolipid biosynthesis.** Serine palmitoyltransferase and 3-KDHS reductase are enzymes responsible for the production and degradation of 3-KDHS, which is the key initial metabolite of sphingolipid biosynthesis. Grey lines are results of 2,000 individual iterations in the large-scale simulation. Red lines are ensemble averages, and dotted blue lines enclose 95% of the results. The figure corresponds to Figure 3 of the main text.


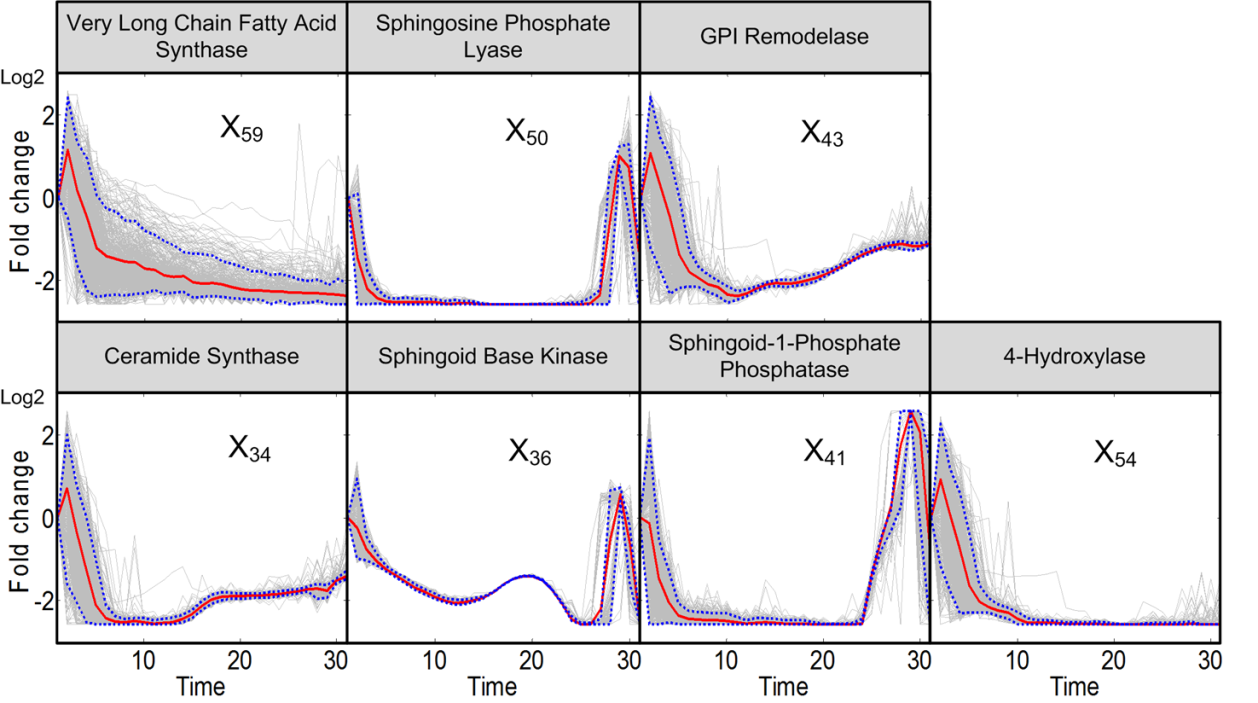


**FIGURE S2. Trends in activities of enzymes in the core region of sphingolipid metabolism.** After an initial spike, all enzyme activities in this region are reduced to almost nil. Grey lines are results of 2,000 individual iterations in the large-scale simulation. Red lines are ensemble averages, and dotted blue lines enclose 95% of the results. The figure corresponds to Figure 4 of the main text.


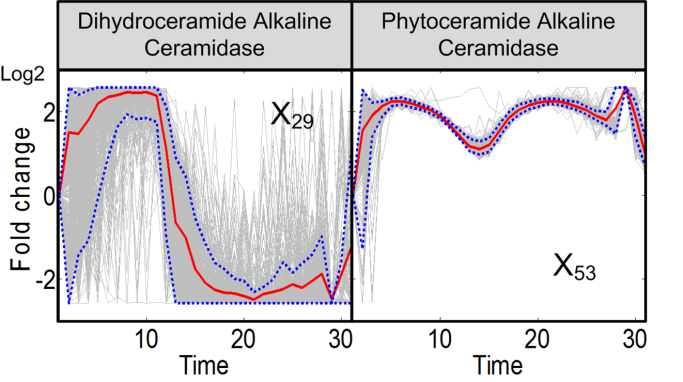


**FIGURE S3. Trends in activities of the two alkaline ceramidases.** Dihydroceramide alkaline ceramidase and phytoceramide alkaline ceramidase, which convert the ceramide form into sphingosines, exhibit distinct activity patterns. Grey lines are results of 2,000 individual iterations in the large-scale simulation. Red lines are ensemble averages, and dotted blue lines enclose 95% of the results. The figure corresponds to Figure 5 of the main text.


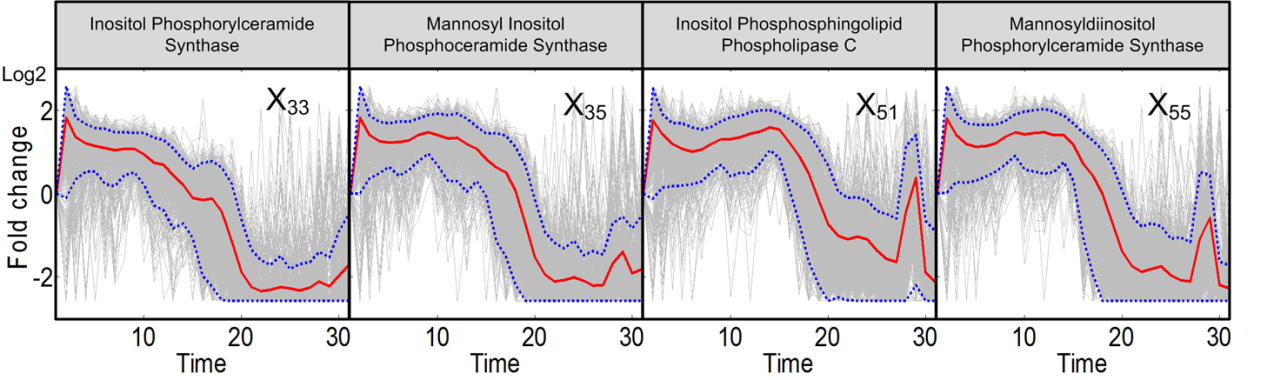


**FIGURE S4. Trends in activities of enzymes associated with complex sphingolipids.** Enzymes interconverting complex sphingolipids are at first hyper-active, but tend to lose most activity between 20 and 30 minutes. Grey lines are results of 2,000 individual iterations in the large-scale simulation. Red lines are ensemble averages, and dotted blue lines enclose 95% of the results. The figure corresponds to Figure 6 of the main text.


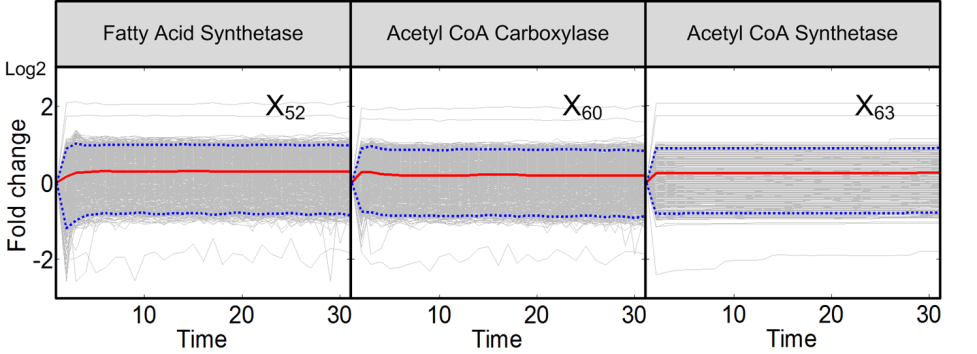


**FIGURE S5. Trends in activities of enzymes associated with fatty acid CoA.** The enzymes shown here are responsible for CoA enlongation. Grey lines are results of 2,000 individual iterations in the large-scale simulation. Red lines are ensemble averages, and dotted blue lines enclose 95% of the results. The figure corresponds to Figure 7 of the main text.


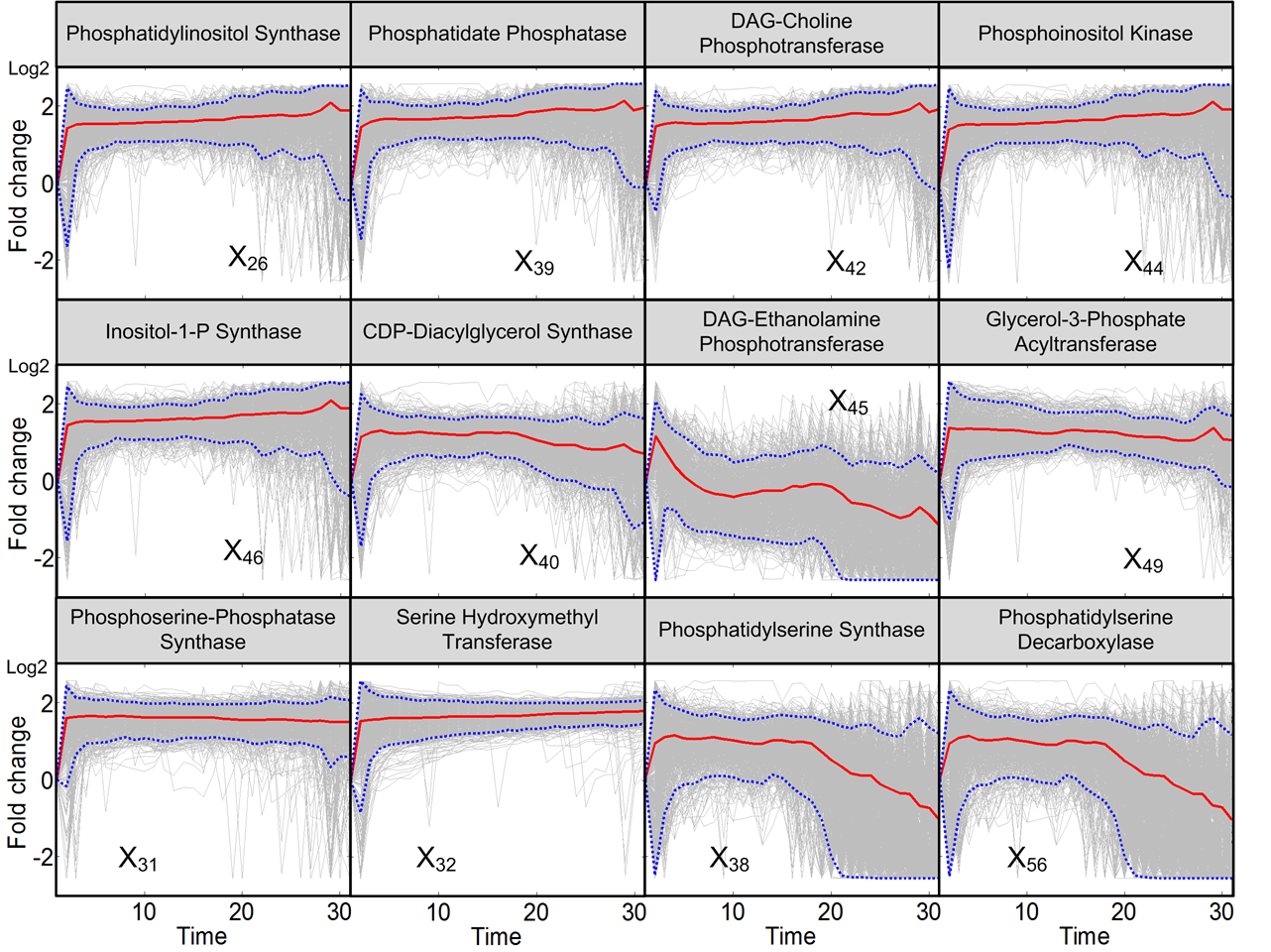


**FIGURE S6. Trends in the remaining enzyme activities.** Activities of enzymes at the periphery of the pathway system are not identifiable, mainly due to insufficient information and the fact that these enzymes are also involved in other pathways. Enzymes in two upper panels are related to the phospholipid metabolism and enzymes in the lower panel are related to serine metabolism. Grey lines are results of 2,000 individual iterations in the large-scale simulation. Red lines are averages, and dotted blue lines enclose 95% of the results. The figure corresponds to Figure 8 of the main text.

***Table of Trends in Enzyme Activities***

Figure 9 of the main text summarizes the results in earlier figures in a visual manner. Table S2 shows a different representation of the same results.

**Table S2: Summary of Identifiable Dynamic Changes in Enzyme Activities in Response to Heat Stress**

All enzyme activities initially climb to different degrees, presumably due to the Arrhenius effect. Subsequently, the trends are strikingly different. Numbers represent approximate fold changes in activities, while arrows and colors indicate the direction of change.

| Enzyme | Time  Var. | 0-5 | | | | 5-10 | 10-15 | | 15-20 | | 20-25 | 25-30 | | | | |
| --- | --- | --- | --- | --- | --- | --- | --- | --- | --- | --- | --- | --- | --- | --- | --- | --- |
| 3-keto-dihydrosphingosine reductase | *X*_27_ | ↑ 2.8 | | | | ↓ 1.1 | ↑ 1.2 | | ↓ 1 | | ↓ 0.5 | ↓ 0.4 | | | | |
| Dihydroceramide aklaline ceramidase | *X*_29_ | ↑ 5.5 | | | | | ↔ 0.3 | | | | | ↑ 0.5 | | | | |
| Inositol phosphorylceramide synthase | *X*_33_ | ↑ 3.5 | | | | ↓ 1 | | | ↓0.3 | | | ↑ 0.4 | | | | |
| Ceramide synthase | *X*_34_ | 0-2.5 | | | 2.5-5 | ↔ 0.3 | | | ↑ 0.4 | | | | | | | |
|  |  | ↑ 1.8 | | | ↓ 0.3 |  |  |  |  |  |  |  |  |  |  |  |
| Mannosyl inositol phosphoceramide synthase | *X*_35_ | 0-2.5 | | | 2.5-5 | ↑ 2.8 | ↓ 0.3 | | | | ↔ 0.3 | | | | | |
|  |  | ↑ 3.5 | | | ↓ 2.5 |  |  |  |  |  |  |  |  |  |  |  |
| Sphingoid base kinase | *X*_36_ | 0-2.5 | | | 2.5-5 | ↔ 0.3 | | | | | | 25-28 | | | 28-30 | |
|  |  | ↑ 1.1 | | | ↓ 0.4 |  |  |  |  |  |  | ↑ 1.8 | | | ↓ 0.3 | |
| Sphingoid 1 phosphate phosphatase | *X*_41_ | 0-2.5 | | | 2.5-5 | ↔ 0.3 | | | | | | 25-29 | | | 29-30 | |
|  |  | ↑ 1.2 | | | ↓ 0.3 |  |  |  |  |  |  | ↑ 6 | | | ↓ 0.5 | |
| GPI remodelase | *X*_43_ | 0-2.5 | | | 2.5-5 | ↓ 0.3 | ↔ 0.3 | | | | ↑ 0.4 | ↑ 0.5 | | | | |
|  |  | ↑ 2.2 | | | ↓ 0.4 |  |  |  |  |  |  |  |  |  |  |  |
| Sphingosine phosphate lyase | *X*_50_ | ↓ 0.2 | | | | ↔ 0.3 | | | | | | 25-28 | | | | 28-30 |
|  |  |  |  |  |  |  |  |  |  |  |  | ↑ 2 | | | | ↓ 0.5 |
| Inositol phosphosphingolipid phospholipase C | *X*_51_ | 0-2 | | 2-5 | | ↑ 2.5 | ↑ 3 | | ↓ 0.5 | | ↓ 0.3 | 25-28 | | | 28-30 | |
|  |  | ↑ 3.4 | | ↓ 2.1 | |  |  |  |  |  |  | ↑ 1 | | | ↓ 0.3 | |
| Phytoceramide alkaline ceramidase | *X*_53_ | ↑ 4.5 | | | | ↓ 3.7 | ↓ 2.2 | | ↑ 4.5 | ↓ 3.7 | | 25-28 | | 28-30 | | |
|  |  |  |  |  |  |  |  |  |  |  |  | ↑ 6 | | ↓ 2 | | |
| 4 hydroxylase | *X*_54_ | 0-2 | | | 2-5 | ↔ 0.3 | | | | | | | | | | |
|  |  | ↑ 2 | | | ↓ 0.3 |  |  |  |  |  |  |  |  |  |  |  |
| Mannosyldiinositol phosphorylceramide synthase | *X*_55_ | 0-2 | | | 2-5 | ↑ 2.7 | 10-13 | 13-15 | ↓ 0.5 | | ↓ 0.3 | 25-28 | 28-30 | | | |
|  |  | ↑ 3.5 | | | ↓ 2.2 |  | ↓ 2.6 | ↓ 1.7 |  |  |  | ↑ 0.6 | ↓ 0.3 | | | |
| Serine palmitoyltransferase | *X*_57_ | 0-2 | 2-5 | | | ↓ 0.3 | ↔ 0.3 | | | | | | | | | |
|  |  | ↑ 2.2 | ↓ 0.5 | | |  |  |  |  |  |  |  |  |  |  |  |
| Very long chain fatty acid synthase | *X*_59_ | 0-2 | 2-5 | | | ↓ 0.3 | ↔ 0.3 | | | | | | | | | |
|  |  | ↑ 2.3 | ↓ 0.5 | | |  |  |  |  |  |  |  |  |  |  |  |

***Estimation of Q_10_ values for Enzymes of the Sphingolipid Pathway***

The “initial jump” of enzyme activities allows us to estimate Q_10_ values for the different enzymes. These values are computed from the definition

$Q_{10}=\left\lceil\frac{R_{1}}{R_{0}} \right\rceil^{\frac{10}{T_{1}-T_{0}}}$.

Here, $R_{0}$ is the base line enzyme activity under optimal conditions (30°C) and $R_{1}$ is the enzyme activity immediately following the temperature change to (39°C); the difference between the two is $T_{1}-T_{0}$. The computed Q_10_ values are summarized as in Table S3. Values were only estimated for identifiable enzymes.

**Table S3: Estimated Q_10_ Values, Based on the Initial Increases in Enzyme Activities**

| **Enzyme** | **Variable** | **Q_10_** |
| --- | --- | --- |
| 3-keto-dihydrosphingosine reductase | *X*_27_ | 3.1394 |
| Dihydroceramide aklaline ceramidase | *X*_29_ | *3.0150* |
| Inositol phosphorylceramide synthase | *X*_33_ | *4.0227* |
| Ceramide synthase | *X*_34_ | *1.9215* |
| Mannosyl inositol phosphoceramide synthase | *X*_35_ | *4.0227* |
| Sphingoid base kinase | *X*_36_ | *1.1117* |
| Sphingoid 1 phosphate phosphatase | *X*_41_ | *1.2246* |
| GPI remodelase | *X*_43_ | *2.4014* |
| Sphingosine phosphate lyase | *X*_50_ | *0.1673* |
| Inositol phosphosphingolipid phospholipase C | *X*_51_ | *3.8952* |
| Phytoceramide alkaline ceramidase | *X*_53_ | *3.5153* |
| Hydroxylase | *X*_54_ | *2.1601* |
| Mannosyldiinositol phosphorylceramide synthase | *X*_55_ | *4.0227* |
| Serine palmitoyltransferase | *X*_57_ | *2.4014* |
| Very long chain fatty acid synthase | *X*_59_ | *2.5230* |

***Assessment of Simulation Results with a “Negative Control”***

The main text described different validation methods for our simulation results. Most importantly, the reconstructed sphingolipid profiles from the means of computed enzyme activities fit the heat stress data well (Figure 1). In addition, we examined the concentrations of members of the IPC family, which were not used as inputs. The simulations indicate that these concentrations should be essentially constant during the heat stress response and, indeed, these results are consistent with the literature [[4](#_ENREF_4)]. The third assessment mentioned, but not detailed in the text, is a “negative control.” We fixed eight key enzymes (*X*_34_, *X*_36_, *X*_41_, *X*_43_, *X*_50_, *X*_54_, *X*_57_ and *X*_59_) at their optimal steady-state values and then performed the same optimization as before. The thus inferred enzyme activities of this “constrained model” do not produce good fits to the sphingolipid heat stress data (Figure S7). Specifically, the sum of squared errors (SSE) for Figure 1 is 5.79×10^-4^, whereas it is 2.79×10^-2^ for Figure S7. In order to assess the quality of fit further, it is useful to display the residual errors of the individual optimizations with the constrained model in comparison to those obtained with the model in the text. Figure S8 clearly demonstrates that the residual errors of the 2,004 original simulations are much lower than those of 200 constrained simulations. In this representation, the X-axis shows the index of each individual simulation and the Y-axis shows the corresponding SSE. Figure S9 shows distributions of the SSEs in the two scenarios. These assessments clearly show that the SSEs for the constrained model are much larger than the corresponding SSEs for the original model in the body of the paper and therefore suggest that the key enzymes in the sphingolipid system must respond to heat stress in a coordinated manner.

One should note that SSEs of individual simulations are smaller than those of the averaged model fit. As mentioned in the text, parameterization with averaged values does not necessarily lead to good fits. In the present case, the averaged model in Figure 1 of the text is visually not all that different from the best individual simulation results (threshold: SSE < 1.25×10^-5^) displayed in Figure S2 (upper panel). The similarity of these fits is shown in Figure S10. The higher SSE of the averaged fit is presumably due to the fact that the plots show fold changes and X6 (PHS-P) has a low steady-state of 0.005, while X3 (DHC) has a substantially higher level (0.0366), but does not change all that much in actual value.”


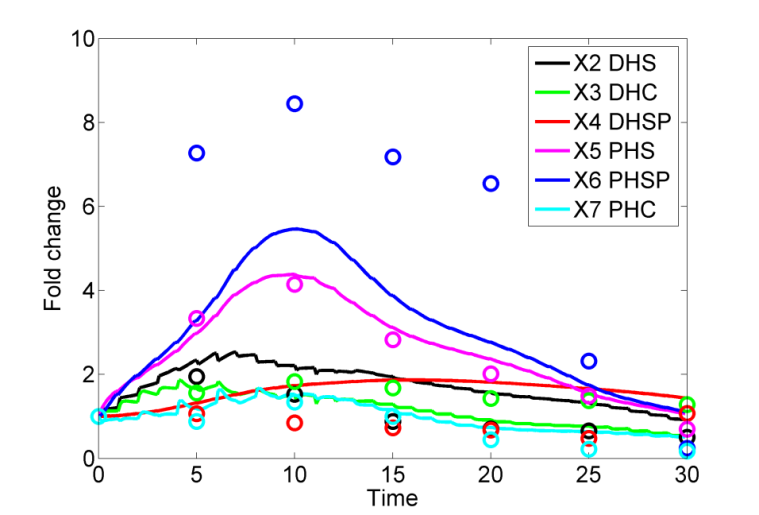


**Figure S7:** When the key enzymes are locked into their normal activity values and all other enzyme activities are allowed to be optimized, the fit of the best model to the experimental data is not very good.


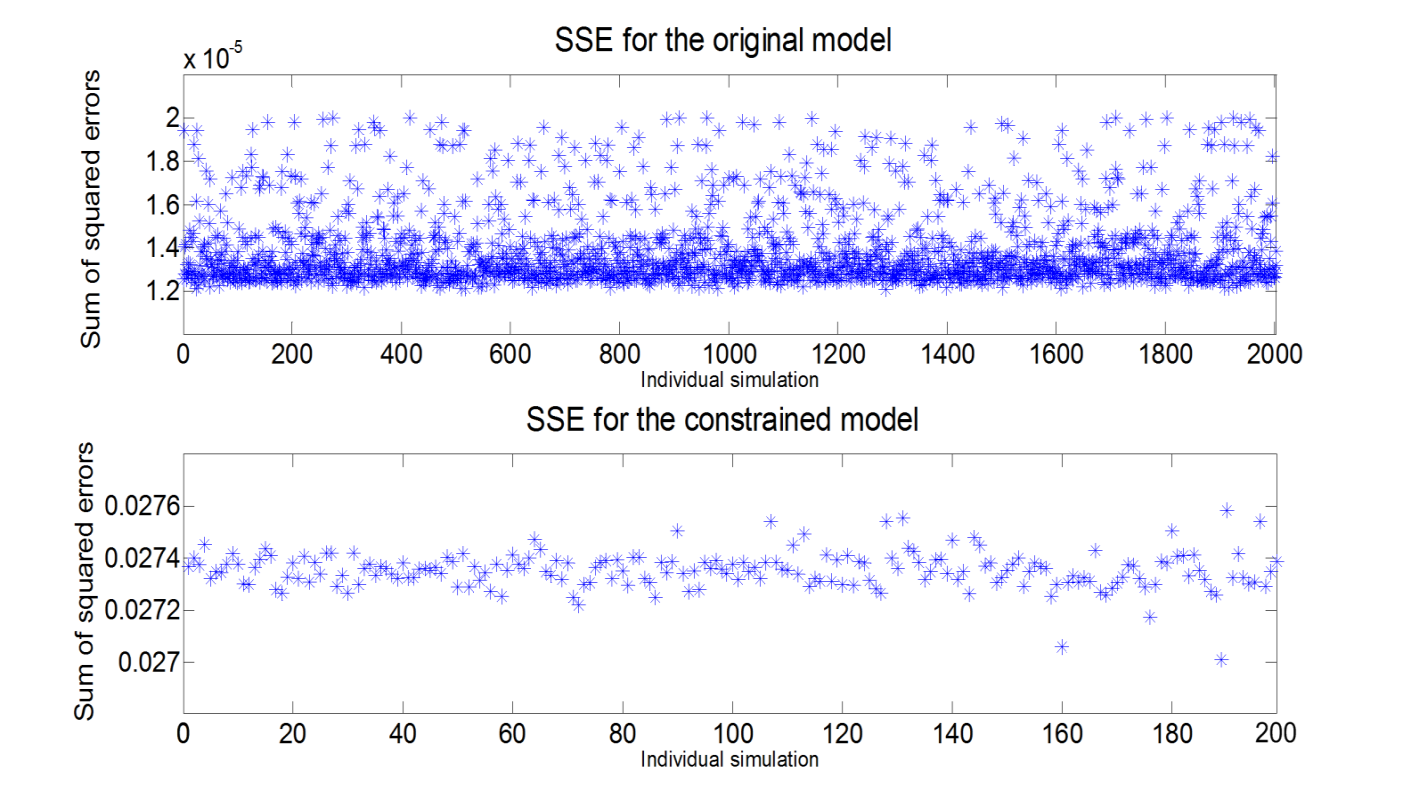


Figure S8. Sums of squared errors for individual optimizations. Upper panel: SSEs for 2,000 simulations with the original model. Lower panel: SSEs for 200 simulations with the constrained model. The *X*-axis shows the index of each individual simulation, while the *Y*-axis shows the corresponding sum of squared errors (SSE); note different scales.


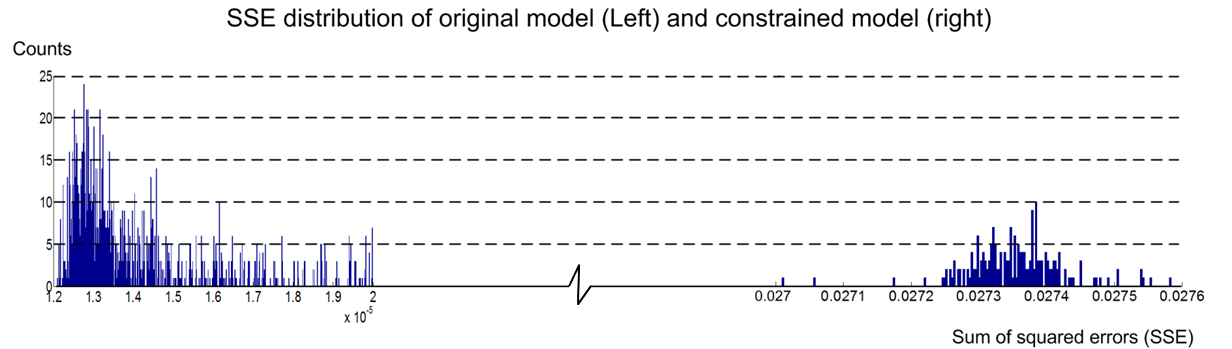


Figure S9. Distributions of sums of squared errors for individual simulations. The distribution on the left contains SSEs for the model in which all enzymes are allowed to change. The distribution on the right contains the corresponding SSE values for the constrained model.


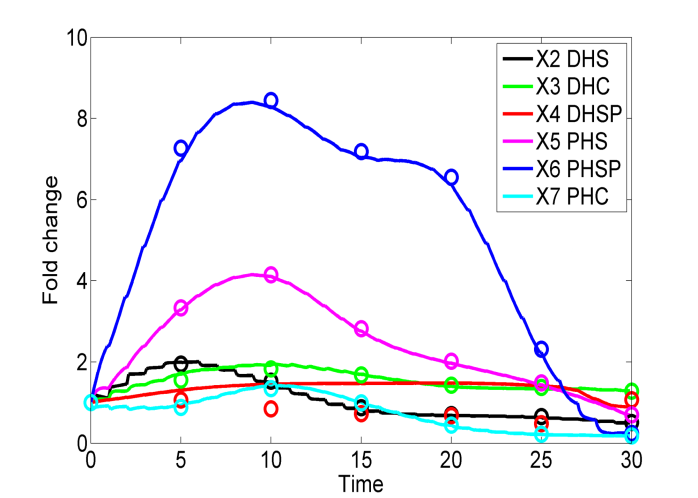

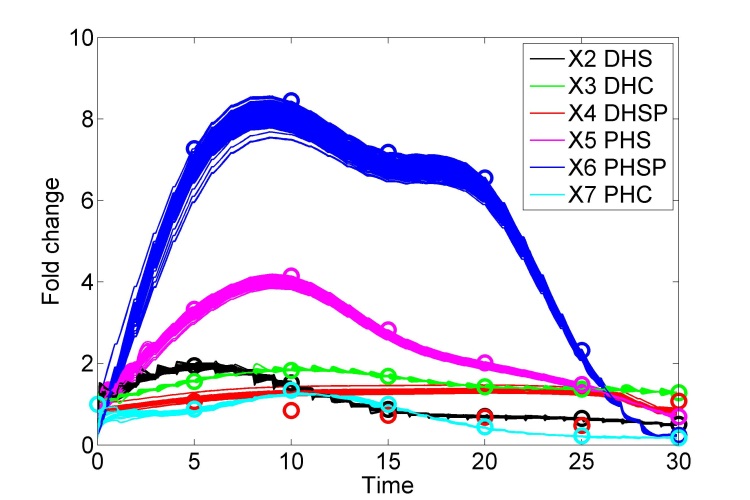


Figure S10. Comparison of data fits. Left panel: Data fitted with the unconstrained averaged model (identical to Figure 1 of the text). Right panel: 179 data fits with individual model simulations that resulted in SSE < 1.25×10^-5^ (*cf.* Figure S8).

***Akaike Information Criterion***

The Akaike Information Criterion (AIC) provides a measure of goodness-of-fit for multi-model inferences. We used this criterion to assess the quality of our model. While we are only using one model, the AIC value result may be helpful for future comparisons with alternative models. The formulation of AIC was adapted from [[5](#_ENREF_5)]. AIC is based on the Kullback-Leibler (K-L) information measure *I*(*f*, *g*) given below:

$$I\left( f,g \right)=\int f\left( x \right)log(\frac{f(x)}{g(x|\theta)})dx$$

Here, *f*(*x*) refers to the true representation the model is supposed to capture and *g*(*x*|θ) is the model. *I*(*f*, *g*) can be written in terms of expectations as

$$I\left( f,g \right)=\int f\left( x \right)log\left( f\left( x \right) \right)dx-\int f\left( x \right)log\left( g\left( x | \theta\right) \right)dx$$

$$=E_{f}\left[ log\left( f\left( x \right) \right) \right]-E_{f}\left[ log\left( g\left( x | \theta\right) \right) \right]$$

Here, the first term refers to the true representation, which however is unknown and therefore replaced with an unknown constant $C= E_{f}\left[ log\left( f\left( x \right) \right) \right]$. The second term requires the estimation of the expectation of *g* given data *y*, $E_{y}E_{x}\left[ log\left( g\left( x | \hat{\theta}(y) \right) \right) \right]$, where $\hat{\theta}(y)$ is the maximum likelihood estimator with respect to *y*.

Akaike ([[6](#_ENREF_6),[7](#_ENREF_7)]) found that the maximum likelihood estimator for $E_{y}E_{x}\left[ log\left( g\left( x | \hat{\theta}(y) \right) \right) \right]$ is biased, but that there is a relationship between the bias (called $K$) and the K-L information, namely:

$$log\left( L\left( \hat{\theta}\left( y \right) \right) \right)-K=C-E_{\hat{\theta}}\left[ I\left( f,\hat{g} \right) \right]$$

Furthermore, the bias *K* is equal to the number of estimated parameters. Akaike thus introduced AIC as

$$AIC=-2log\left( L\left( \hat{\theta} | y \right) \right)+2K$$

For least square estimation, this definition reduces to

$$AIC=nlog\left( \sigma^{2} \right)+2K$$

Where $n$ is the number of data, $\sigma^{2}:\frac{\sum_{i=1}^{n} {(\varepsilon_{i})}^{2}}{n}$ and *ɛ_i_* is the sum of squared errors for each sample. To extend the applicability of AIC further, Akaike introduced AIC_c_$\mathrm{AIC}_{c}$ to deal with small samples, which are defined here as $\frac{sample size n}{\# paramter K}$ < 40. The result is:

${AIC}_{c}=AIC+\frac{2K(K+1)}{n-K-1}$.

We obtained the AIC and AICc values for our 4414 models by using the definitions given above. The parameter *n* represents the number ofdata, which in our case is $n=30*6=180$. $\sigma^{2}$ is the mean sum of squared errors (SSE) for all simulations. As we have 30 points to fit in each simulation, $\sigma^{2}$ can be computed as

$$\sigma^{2}= \frac{\sum_{i=1}^{n} (\sum_{j=1}^{30} {(X(j)-Data(j))}^{2})}{n}$$

Here, *X* is the vector of six sphingolipids and *Data* represents the vector with the corresponding smoothed data.

The main text shows the results of 2004 models selected from a much larger pool of 4144 initial models, based on the sum of squared errors (SSEs). Computing the average of these 2004 models, we further provided a validation for the use of the averaged model for later interpretations of trends in enzyme activities, by comparing the observed heat stress data with the heat stress profiles computed with this model. This comparison yielded a good fit. To test the validity of the 2004 models and their average further, we performed a comparison between the results using either the SSE or the AICc criterion for model selection. Specifically, we computed AICc values for all 4144 models and binned them in a histogram (Figure S11). This histogram very nicely shows that 2018 models (left-most column) are superior to the others. Comparing this set of models with the set identified by SSEs (2004 models), we found that 99.35% (1991) of the 2004 SSE models simultaneously also satisfied the AICc criterion. Thus, we may use either set of models to make inferences regarding enzyme activities.


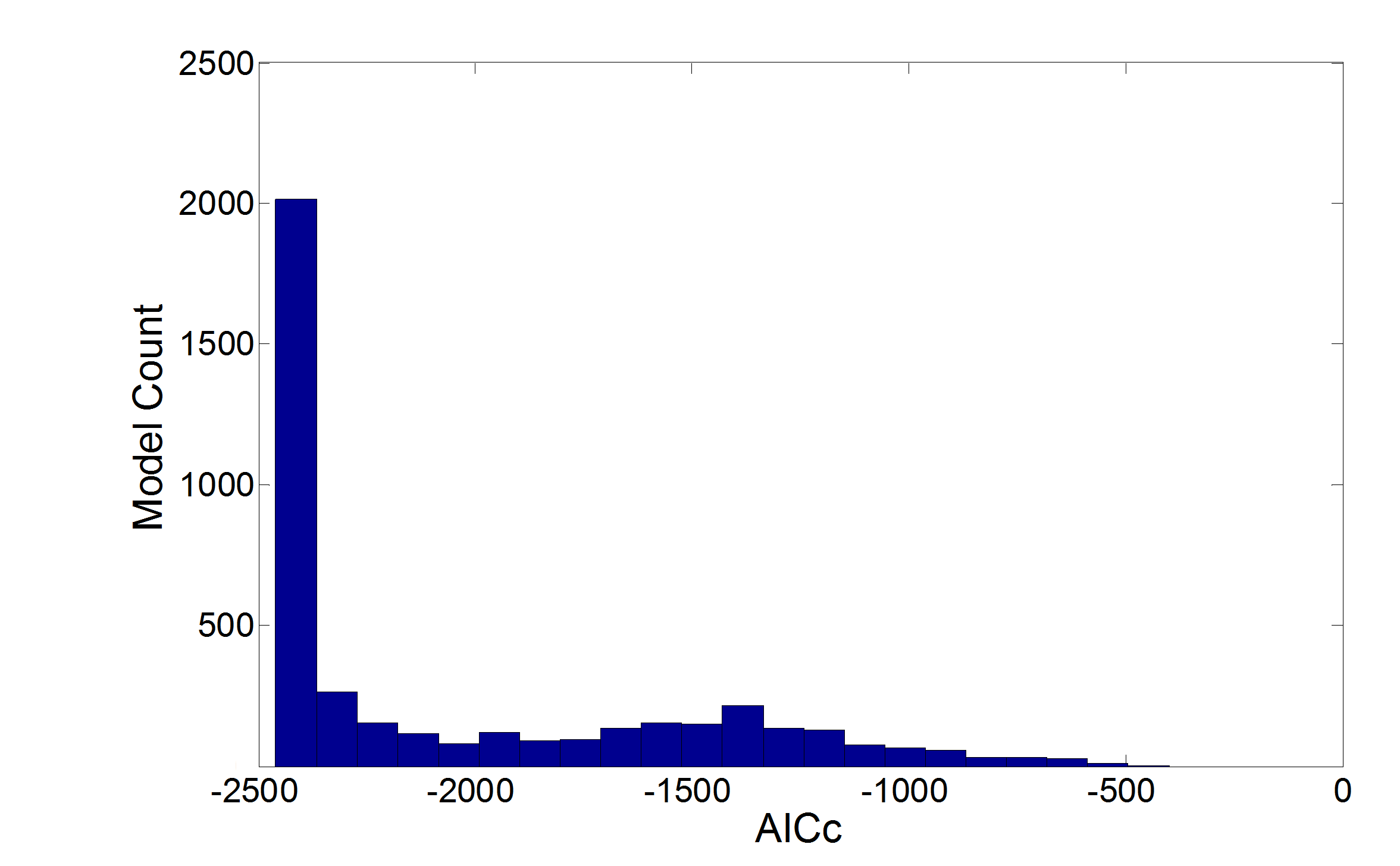


Figure S11: The histogram of AICc values of the 4144 initial models clearly indicates that the 2018 models in the left-most column are superior to all other parameterizations. 99.35% (1991) of the 2004 models identified by SSE fall into this column, thereby demonstrating very strong consistency between the two measures of quality.

***References***

1. Alvarez-Vasquez F, Sims KJ, Cowart LA, Okamoto Y, Voit EO, et al. (2005) Simulation and validation of modelled sphingolipid metabolism in *Saccharomyces cerevisiae*. Nature 433: 425-430.

2. Alvarez-Vasquez F, Sims KJ, Hannun YA, Voit EO (2004) Integration of kinetic information on yeast sphingolipid metabolism in dynamical pathway models. Journal of Theoretical Biology 226: 265-291.

3. Alvarez-Vasquez F, Sims K, Voit E, Hannun Y (2007) Coordination of the dynamics of yeast sphingolipid metabolism during the diauxic shift. Theoretical Biology and Medical Modelling 4: 42.

4. Jenkins GM, Richards A, Wahl T, Mao C, Obeid L, et al. (1997) Involvement of yeast sphingolipids in the heat stress response of *Saccharomyces cerevisiae*. Journal of Biological Chemistry 272: 32566-32572.

5. Burnham KP, Anderson DR (2002) Model Selection and Multi-Model Inference. New York: Springer-Verlag.

6. Akaike H (1973) Information theory as an extension of the maximum likelihood principle. In: Petrov BN, Budapest FC, editors. Second International Symposium on Information Theory. pp. 267-281.

7. Akaike H (1974) A new look at the statistical model identiﬁcation. IEEE Transactions on Automatic Control: AC-19:716-723.
